# Core transcriptional regulatory circuitry molecule ZNF217 promotes AML cell proliferation by up-regulating MYB

**DOI:** 10.7150/ijbs.103211

**Published:** 2025-02-18

**Authors:** Bi Zhou, Fang Fang, YongPing Zhang, ZhiHeng Li, YiXin Hu, Yan Li, WanYan Jiao, YuMeng Wu, XiaoMei Wan, Ying Yang, FenLi Zhang, Ling Xu, TongTing Ji, Jian Pan, ShaoYan Hu

**Affiliations:** 1 Children's Hospital of Soochow University, Suzhou, 215003, China.; 2 Dept. of Pediatric, Suzhou Hospital of AnHui Medical University, Suzhou, 234000, China.; 3 Institute of Pediatric Research, Children's Hospital of Soochow University, Suzhou 215003, China.; 4 Pediatric Hematology & Oncology Key Laboratory of Higher Education Institutions in Jiangsu Province, Jiangsu, China.; 5 Dept. of Hematology, Children's Hospital of Soochow University, Suzhou, 215003, China.; 6 Dept. of Pediatric, The Affiliated Hospital of Xuzhou Medical University, Xuzhou, 221000, China.; 7 Dept. of Pediatric, Yancheng Third People' Hospital, YanCheng, 224000, China.; 8 Dept. of Pediatric, The First Affiliated Hospital of Bengbu Medical College, Bengbu, 233004, China.; 9 Dept. of Pediatric, The First Affiliated Hospital of Wannan Medical College, Wuhu, 234100, China.; 10 Clinical Medicine, Guizhou Medical University, Guiyang 550000, China.

**Keywords:** Pediatric, AML, ZNF217, MYB, Super enhancers, CRC

## Abstract

Leukemia is characterized by multiple rearrangements of signal transduction genes and overexpression of nonmutated genes, such as transcription factors (TFs) genes. Super-enhancers (SEs) are prevalent in human cancers and are associated with the accumulation of numerous core TFs. SEs drive the expression of core TF genes by delivering robust transcriptional activation signals. Additionally, core TFs sustain the stability and activity of SEs through mutual auto-regulation loops, creating a positive feedback loop known as the Core Transcriptional Regulation Circuit (CRC).

Using ChIP-seq data, we identified the involvement of the SE-related gene ZNF217 in acute myeloid leukemia (AML), in which its functional role and underlying mechanism remain unclear. We demonstrated that ZNF217, ELF1, MEF2D, RUNX2, and FOXP1 are likely integral components of the AML CRC through various experimental techniques, including CUT&Tag, short hairpin RNA (shRNA) transduction, and Luciferase reporter assays. Notably, ZNF217 was determined to be indispensable for the proliferation and viability of AML cells both *in vitro* and *in vivo*. Subsequent analysis of RNA-seq and CUT&Tag results identified MYB as a key direct target of ZNF217. Overall, our research highlights ZNF217 as a critical oncogene in AML and offers new insights into the transcriptional regulatory mechanisms at play in AML.

## Introduction

Transcriptional abnormalities are a key aspect of cancer biology and involve changes in both protein-coding genes and noncoding regulatory elements. This important feature is primarily mediated by large regulatory elements, such as SEs, which act as essential cis-acting elements. Compared to ordinary enhancers, SEs are longer, contain a higher density of TFs, and interact more strongly with the transcriptional machinery. These characteristics enhance the regulatory role of TFs in determining cellular identity and fate, significantly impacting various cell types. SEs regulate the expression of cancer-related genes by recruiting numerous core TFs, thereby forming robust transcriptional regulatory complexes. Certain core TFs form CRCs that maintain the stability and activity of SEs, creating a positive feedback loop. This synergy enhances transcriptional activation efficiency and ensures both the specificity and precision of gene expression 1, 2.

The formation of oncogenic SEs drives tumorigenesis, a complex process where specific body cells transform into tumor cells. Acquired SEs in tumor cells specifically increase the expression of oncogenes. This increase leads to the dysregulation of signaling pathways. In multiple myeloma (MM) cells, these oncogenic SEs are closely linked to genes that regulate survival specific to their lineage, such as IGLL5, MYC, IRF4, and XBP1, promoting tumorigenesis by enhancing oncogene expression. The transcription of SE-regulated genes involves crucial mediators such as the Mediator complex, BRD4, and CDK. JQ1, a BRD4 inhibitor, can significantly decrease the expression of SE-associated genes in MM cells 3. Notably, oncogene expression is often elevated in cancer cells through the action of SEs. It has been reported that the binding sites of TCF4, a crucial transcription factor intrinsic to the Wnt signaling pathway, are abundant in colorectal cancer-associated SEs but absent in DNA in normal colorectal cells 4. In the context of T-cell acute lymphoblastic leukemia (T-ALL), the overexpression of TAL1 is closely associated with the formation of SEs. TAL1, located upstream of SEs, carries a short heterozygous mutation that results in the formation of an SE capable of binding to the MYB transcription factor within a noncoding region. The interaction between MYB and the SEs forms a positive feedback loop, promoting the proliferation of T-ALL 5. Researchers identified a specific set of SEs by carefully mapping the enhancer landscape in each subgroup. The aforementioned surrogate endpoints, i.e., SEs, have the ability to predict subgroup status autonomously, exhibit a correlation with tumor heterogeneity, and bring attention to potential treatment vulnerabilities 6. Therefore, targeting the formation and activation of oncogenic SEs is increasingly being recognized as a potent emerging strategy in cancer therapy.

Our group identified the cancer-promoting roles of SE-associated genes, such as LYL1, LMO2, INSM2, and FYB1 in tumor development 7-11. Empirical evidence suggests that BRD4 inhibitors significantly reduce neuroblastoma and AML cell proliferation both *in vitro* and *ex vivo* 12, 13. However, studies on SEs in AML are scarce. In this study, our analysis uveiled the SE landscape within the samples of 11 cases of pediatric AML patients and 7 AML cell lines, identifying regions where SEs occurred in at least 80% of the samples examined. Significantly, the analysis reveals that ZNF217 occupies the top position in terms of significance and is a key component of the AML core regulatory circuit. ZNF217, located on chromosome 20q13, is a member of the zinc finger transcription family and plays a crucial role in regulating gene expression in eukaryotes. Research indicates that ZNF217 is involved in various human cancers, including breast cancer 14^,^ ovarian cancer 15^,^ stomach cancer 16, colon cancer 17, prostate cancer 18, glioblastoma 19, lung cancer 20, and lymphoma 21, as well as with the precancerous condition Barrett's esophagus 22. Aberrant amplification of ZNF217 has been observed in various cancers and precancerous conditions. This amplification is associated with an increased risk of cancer and drug resistance 23. Furthermore, we explored the functional role of ZNF217 in AML cell proliferation, cell cycle regulation, and apoptosis, as well as the upstream and downstream regulatory mechanisms within the context of AML.

## Materials and Methods

### Clinical samples and cell sorting

We compared ZNF217 expression between primary AML patients and normal HSPCs using fresh whole bone marrow (BM) samples collected at diagnosis from the Children's Hospital of Soochow University. We also obtained fresh BM samples from healthy donors at the Children's Hospital of Soochow University. Using a flow cytometer, we isolated the CD34^+^CD38^-^ cell population from the BM of healthy children. Next, we stained BMMCs with the following antibodies: CD34^-^APC, CD45-PE, and CD38-FITC. CD34^+^CD38^-^ cells were sorted and analyzed using FACSAria III and FACSAria SORP cell sorters (BD Biosciences) with FlowJo Software (V10). The RNA-Seq data from 9 AML pediatric samples and 6 normal control samples have been uploaded to the GEO database (GSE190775 and GEO260775).

Additionally, we obtained BM specimens from three pediatric AML patients who were newly diagnosed and undergoing treatment at our institution. Simultaneously, BM samples from 5 healthy volunteer children (HSPCs isolated by flow cytometry) were obtained as controls. All specimens were processed within 24 hours, with total RNA extraction performed and stored at -80°C. Then, reverse transcription and qRT-PCR analyses were uniformly performed on the 8 samples. All participants in this study provided written informed consent for the sample collection and detailed analyses.

### Cell lines and culture

We obtained several human AML cell lines, including HL60, U937, MV4-11, Kasumi-1, HEL, MOLM13, MOLM16, NB4, and K562, from the National Collection of Authenticated Cell Cultures located in Shanghai, China. The cells were cultured in RPMI 1640 medium, adding 10% fetal bovine serum (VivaCell) and 1% penicillin-streptomycin (Beyotime). The culture medium for HSPCs was supplemented with 100 ng/mL FLT3L, 20 ng/mL SCF, 20 ng/mL IL-3, and 20 ng/mL TPO. The cells were cultured at 37 ℃ in an atmosphere containing 5% carbon dioxide. Additionally, we acquired 293FT cells from the cell repository at the Chinese Academy of Sciences and cultured them in complete DMEM growth medium (VivaCell). All cell lines were subjected to routine testing using a MycoAlert Kit to confirm the lack of contamination with Mycoplasma.

### Establishment of stable and inducible cell lines

We used the pLKO.1-puro backbone (IGE Biotechnology Limited) to create lentiviral vectors with specific shRNA sequences that target genes like ZNF217, ELF1, MEF2D, RUNX2, and FOXP1, aiming to establish stable and inducible cell lines.

We cotransfected the targeted plasmids into 293FT cells using the packaging plasmid psPAX2 and the envelope plasmid pMD2.G (pMD2.G:#12259; psPAX2: #12260; Cambridge, MA) along with polyethylenimine (PEI) reagent (49553-93-7, Sigma-Aldrich) at a ratio of 4:3:1. After a 6-hour incubation, the viral supernatant was harvested and filtered through a 0.45 µm filter. We performed lentiviral transduction of AML cells in the presence of 1 µg/mL polybrene (Sigma-Aldrich) to enhance the efficiency.

We generated stable gene knockout cell lines using the CRISPR/Cas9 system by first transducing lentiviral vectors carrying the Cas9 gene into U937 and MV4-11 cells, followed by selection of stable transductants with puromycin. We cloned single guide RNAs (sgRNAs) that target ZNF217 and inserted them into the Lenti-CRISPR plasmid provided by GeneChem (China). Cas9-expressing cells were lentivirally transduced with either sgRNA-ZNF217 or a nontargeting control sgRNA (sgNC). Lentivirus preparation was conducted by GeneChem using a previously described method.

To achieve stable overexpression of ZNF217, the full-length wild-type ZNF217 gene fused to FLAG-tagged sequences or nontargeting control sequences was inserted into the Ubi-MCS-3FLAG-CBh-gcGFP-IRES-puromycin plasmid. The lentivirus used for ZNF217 overexpression was also prepared by GeneChem.

The target sequence information is shown in **Table S1**.

### Detection of ZNF217 expression in HSPCs using flow cytometry

After sorting HSPCs with flow cytometry, we infected the cells with lentivirus to knock down ZNF217, as described in the “Establishment of Stable and Inducible Cell Lines” section. Next, we followed the flow cytometry intracellular staining protocol (https://assets.thermofisher.com/TFS-Assets/BID/Technical-Notes/flow-intracellular-staining-en.pdf) and used ZNF217 antibody along with FITC-conjugated secondary antibody (ThermoFisher, A-11034) to simultaneously detect ZNF217 protein expression levels in HSPCs.

### Cell proliferation assay

Cell viability was assessed with a Cell Counting Kit-8 (CCK-8; K1018, APExBIO) to determine the health of the cells. Each group of cells was placed in a 96-well plate, with 2000 cells per well. A total of 20 µl of CCK-8 reagent was added to each well during the initial cell seeding and again on Days 2, 4, and 6. Absorbance was measured at a wavelength of 450 nm using a Bio-Rad microplate reader to evaluate the cell proliferation rate.

### Soft agar colony formation assay

We prepared a mixture of 1.2% agarose (A9045-10G, SIGMA) and 2× RPMI 1640 medium (YC-1010, YuChun), supplemented with 20% FBS, penicillin, and streptomycin, for the soft agar colony formation assays. We prepared the bottom gel layer by mixing equal parts of 1.2% agarose gel and medium, and the upper gel layer by mixing equal parts of 0.7% agarose gel and medium. After the bottom gel layer solidified, we added the top gel layer along with the cells. The plate was then incubated at 37 ºC with 5% CO2. We added fresh culture medium every three days. Two to three weeks later, we used 4% paraformaldehyde (P0099, Beyotime) and Giemsa (C0131, Beyotime) to fix and stain the cells, respectively, and then counted the colonies.

### Cell cycle analysis

Cells were harvested and washed with cold PBS. They were then fixed overnight in 75% ethanol. Next, the cell cycle distribution was assessed using the Cell Cycle and Apoptosis Analysis Kit (C1052, Beyotime) following the manufacturer's instructions. Flow cytometry was performed with a Gallios™ flow cytometer (Beckman) to determine the cell cycle distribution.

### Cellular apoptosis analysis

After harvesting, cells were placed in cold PBS and then resuspended in a binding buffer. Next, for apoptosis analysis, the cells were stained with a solution containing fluorescein isothiocyanate (FITC)-conjugated Annexin V and propidium iodide (PI) (556420, BD). The stained cells were then analyzed using a Gallios™ flow cytometer (Beckman).

### Dual-luciferase reporter assay

The ZNF217 and RUNX1 SEs were divided into distinct segments. The experimental procedure involved transfecting MV4-11 cells with each segment. Additionally, 0.05 µg of Renilla luciferase plasmid was included as a normalization control. We performed transfection using 0.5 µg of genomic material from each segment. After a 48-hour transfection period, we assessed luciferase activity using the Dual-Luciferase Reporter Assay System (E1910, Promega). This system can simultaneously measure both firefly and Renilla luciferase activity. To account for variations in enhancer segments among the transfected cells, we normalized the calculated ratio of firefly to Renilla luciferase activity. Sequence information for these enhancers can be found in **Table S2**.

### *In vivo* experiments

Experiments were conducted using NSG mice aged 4 to 5 weeks (Shanghai Model Organism). Mice were randomly assigned to different groups and injected with U937 cells expressing either sh-NC (negative control) or sh-ZNF217. On Days 12, 15, 18, and 21 after cell administration, mice with neoplasms received intraperitoneal injections of D-luciferin sodium salt (115144-35-9, GoldBio). Thirty minutes later, under anesthesia, the mice were subjected to *in vivo* imaging using a Berthold imaging apparatus (Berthold, Germany). After euthanasia by CO2 inhalation, we collected bone, hepatic, and splenic tissue samples for further experimentation. This included flow cytometric analysis using CD45 detection, hematoxylin and eosin staining, and immunohistochemical analyses. Antibodies specific for CD45, Ki67, C-MYC, and ZNF217 were used according to the instructions.

### RNA extraction and qRT-PCR

Total RNA was extracted from cellular specimens using the FastPure Cell/Tissue Total RNA Isolation Kit V2 (RC112-01, Vazyme) according to the manufacturer's instructions. Each sample contained 1500 ng of RNA, which was subsequently subjected to reverse transcription to synthesize cDNA. The experimental procedure utilized 200 units of M-MLV reverse transcriptase (Promega), 20 units of RNase inhibitor (Thermo Fisher), and 500 nanograms of random primers (Promega) with an ABI PCR apparatus (Applied Biosystems, Thermo Fisher). Next, quantitative reverse transcription PCR (qRT-PCR) analyses were performed using a LightCycler 480 Real-Time System (Roche). The expression levels of the target genes were normalized against GAPDH levels. The primer sequences used in this study are provided in **Table S3**.

### Western blot analysis (WB)

Protein lysates underwent electrophoretic separation using precast protein gels with a 4%-20% gradient (GenScript). The separated proteins were then transferred to polyvinylidene fluoride (PVDF) membranes (Merck Millipore, Burlington) with a pore size of 0.45 µm. Next, the membranes were blocked at room temperature with a solution of 5% skim milk in TBST (1× TBS, 0.1% Tween 20) for 1.5 hours. Then, horseradish peroxidase-conjugated secondary antibodies (Proteintech) were applied to the membranes, and protein bands were visualized using ultrasensitive ECL reagent (Thermo Fisher). The protein bands were analyzed for expression using the LAS 4010 imaging system (Cytiva). A list of antibodies used in this study can be found in **Table S4**.

### RNA sequencing and data processing

RNA-Seq analysis was performed by Novogene Bioinformatics Technology Co., Ltd. (China). First, total RNA underwent reverse transcription to create cDNA, which was then used to construct a sequencing library. The library was sequenced on appropriate platforms. The raw reads were filtered to remove low-quality reads, adapters, and other artifacts, yielding clean reads. The clean reads were subsequently matched to a reference genome using the HISAT framework to ascertain their mapping positions. Gene expression was quantified using the fragments per million (FPM) metric. Differentially expressed genes (DEGs) were identified through DESeq2 analysis, applying the criteria |log2FoldChange| > 0.5 and p < 0.05. Enrichment analysis was performed using GSEA software, developed jointly by UC San Diego and the Broad Institute, to better understand the biological processes associated with the DEGs. This included: GSE190775: 9 AML samples RNAseq, Illumina NovaSeq 6000 (Homo sapiens); GSE260775: 6 Healthy HSPCs RNAseq, Illumina NovaSeq 6000 (Homo sapiens); GSE246197: 2 MV4-11 samples with no treatment and 2 MV4-11 samples with ZNF217 knockdown RNAseq, Illumina NovaSeq 6000 (Homo sapiens); GSE246199: 2 U937 samples with no treatment and 2 U937 samples with ZNF217 knockdown RNAseq, Illumina NovaSeq 6000 (Homo sapiens).

### CUT&Tag

Samples from 1x10^6 cells were first incubated and activated with ConA beads, followed by an overnight incubation with a secondary antibody diluted to 1:100. Next, Pa/g-Tnp was added at room temperature. The DNA in the sample was then fragmented and extracted for sequencing. The primary antibodies used in these assays included anti-ZNF217, anti-ELF1, anti-MEF2D, anti-FOXP1, anti-RUNX2, anti-LDB1 and IgG. Using Bowtie2 (v 2.4.4), the raw CUT&Tag data were aligned to the UCSC hg38 reference genome with the following parameters: -p 4 -q -x. Regions of interest, represented as peaks, were identified using MACS3 (v3.0) with these parameters: -f BAMPE, -g hs, -B, -q 0.01, and --SPMR. The antibodies used in this study are listed in **Table S4**.

### Coimmunoprecipitation (Co-IP)

After transducing MV4-11 cells with the ZNF217-FLAG-overexpressing lentiviral vector, the cells were lysed in a buffer containing NP-40 (P0013F, Beyotime) and a 100× protease inhibitor. The supernatant was collected by centrifugation. A portion of the supernatant was mixed with 5x protein loading buffer, heated to 100°C, and designated as the input sample. The remaining supernatant was divided. Each portion was incubated separately with protein A/G beads (B23202, Bimake) and either an anti-flag antibody or IgG. The samples were then washed three times. They were lysed in lysis buffer, resuspended in 1x protein loading buffer, and subsequently used for Western blot analysis. Specific immunoprecipitation reactions were performed using anti-ZNF217/Flag antibodies to isolate protein complexes, which were subsequently detected via Western blot analysis. The antibodies used in this study are listed in **Table S4**.

### ChIP-Seq data collection and analysis

In the present study, raw data from our previous ChIP-Seq H3K27ac datasets for 11 case AML samples (GSE188605 7) and public ChIP-Seq datasets for AML cell lines (H3K27ac dataset for MV4-11: GSE80779 24; H3K27ac dataset for THP-1: GSE123872 25; H3K27ac dataset for HL-60: GSE106359 26; H3K27ac dataset for MOLM-13: GSE80779 24; H3K27ac dataset for MOLM-14: GSE65161 27 and H3K27ac dataset for OCI-AML3: GSE101821 28 were aligned to UCSC hg38 (the reference genome) with Bowtie2 (v 2.3.5) 29 with the parameters -p 4 -q -x. Peaks were called with MACS2 (v2.0.9) with the parameters -g hs -n test -B -q 0.01 30. The bigwig files for these datasets were subsequently visualized using Integrative Genomics Viewer (IGV) and the WashU tool (http://epigenomegateway.wustl.edu/browser/) 31. Then, we identified SEs by the ROSE (Rank Order of Super Enhancers) method with the parameters -s 12500 -t 2000 (-s, stitching distance; -t, TSS exclusion zone size) 32.

### Public Hi-C data collection and analysis

Public HiC dataset of MV4-11 cell line (GSE147123) 33 was viewed using the WashU Epigenome Browser (http://epigenomegateway.wustl.edu/browser/) 34.

### Establish an AML CRC model

Self-regulated TF was defined, if a SE-associated TF had more than two binding motifs in its extended SE region. We analyzed the transcriptional connectivity of TFs using a CRC model 35. CRC modeling process is as follows: DNA binding motifs for TFs were obtained using the TRANSFAC database and MEME suite. ROSE-identified SE regions were extended 500 bp on both sides, and then motif scanning was performed using FIMO. Self-regulated TF was defined, if a SE-associated TF had more than two binding motifs in its extended SE region. All possible CRCs were then constructed and scored in a given sample. For each candidate CRC, its score was calculated by dividing the total occurrence times of core TFs in all possible circuitries by the number of core TFs in that circuitry.

### Analysis of target gene expression and survival

We used the Sangerbox 3.0 online database (http://sangerbox.com/home.html) to analyze the expression of the target gene in patients with AML and its relationship to prognosis. Sangerbox 3.0 is a user-friendly pan-cancer analysis tool that utilizes standardized datasets from UCSC (https://xenabrowser.net/), including TCGA, TARGET, and GTEx, covering a total of 19,131 samples and 60,499 genes (PANCAN). Each expression value was log2-transformed (X+0.001). Differential expression between normal and AML samples was assessed using R software (version 3.6.4), and statistical significance was determined using the unpaired Wilcoxon Rank Sum and Signed Rank Tests.

Sangerbox Tools also collected follow-up survival data from TARGET through the UCSC Cancer Browser (https://xenabrowser.net/datapages/), excluding samples with a follow-up duration of less than 30 days. Expression values were log2-transformed (X+0.001), and we subsequently acquired the TARGET-AML expression data and corresponding overall survival (OS) data. Patients were categorized into high and low expression groups based on the median expression value of the target gene, and survival differences between the two groups were compared. The survival differences were analyzed using the survival package in R, employing the survfit function, and the log-rank test was used to evaluate prognostic differences.

Expression values and prognostic data for ZNF217 and MYB in both AML patients and normal controls are downloaded from Sangerbox 3.0 online database, and provided in **Table S5-Table S6**.

### Statistical analysis

Each experiment was conducted independently at least three times, and we provide representative images. We present the experimental results as the mean ± standard deviation (SD) from representative experiments. We used a two-tailed, unpaired Student's t-test for comparisons between two groups. For comparisons among multiple groups, we applied one-way analysis of variance (ANOVA) followed by the Sidak multiple comparison test. We considered statistical significance for P values less than 0.1, denoting it as follows: *P < 0.05, **P < 0.01, and ***P < 0.001. All the statistical analyses and graphing were carried out using GraphPad Prism 9.0 (GraphPad Software Inc., La Jolla, CA, USA) and FigDraw.

## Results

### Identifying SE-related genes in AML

**Figure 1:**
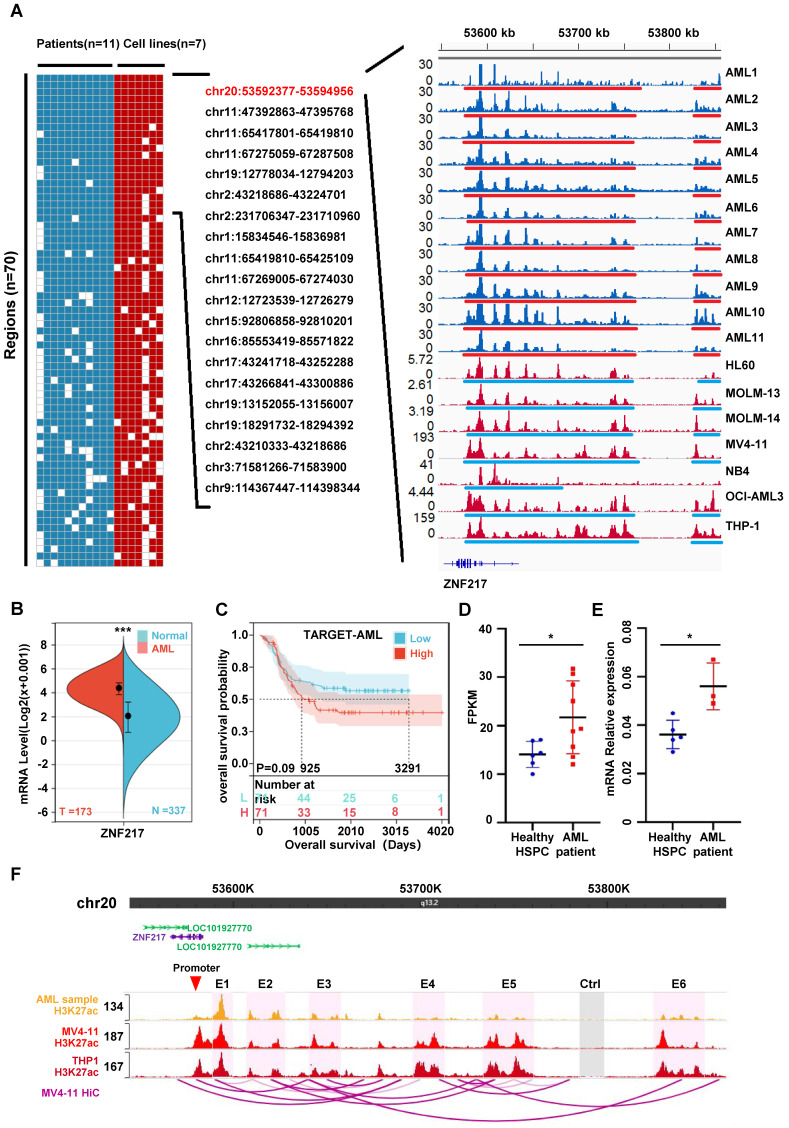
** ZNF217, a super-enhancer-regulated gene, exhibits high expression in AML and is correlated with an unfavorable prognosis. A.** A heatmap was generated to visualize the occurrence (1 or 0) of SEs in AML cell strains and patient samples. The analysis included samples with a frequency exceeding 80% and fragment lengths greater than 2500bp. The H3K27ac activity profile of SE-driven oncogenic genes on chr20:53592377-53594956, which ranked first, was displayed in 11 AML patient samples (blue tracks) and 7 AML cell lines (red tracks) on the right side. SE regions are indicated by underlines. **B.** The ZNF217 expression is significantly upregulated in AML. **C.** Kaplan-Meier curves were employed to analyze the overall survival probability of AML patients based on ZNF217 expression. **D.** RNA-Seq reveals a significant elevation in ZNF217 mRNA expression within bone marrow cells from pediatric AML patients compared to that in healthy children's bone marrow HSPCs. **E.** qRT-PCR reveals a significant elevation in ZNF217 mRNA expression within bone marrow cells from pediatric AML patients compared to that in healthy children's bone marrow HSPCs. **F.** Interactions between the enhancers and promoter region of ZNF217 were observed in MV4-11 and THP1 cells.

Drawing upon our previous study and leveraging public databases, we conducted a reanalysis of the chip-seq data from 11 cases of AML patients 7 and 7 AML cell lines (HL60 26, MOLM-13 24, MOLM-14 27, MV4-11 24, NB4 7, OCI-AML3 28 and THP-1 25). Our analysis uveiled the SE landscape within these 18 samples, identifying regions where SEs occurred in at least 80% of the samples examined. Significantly, the analysis reveals that chr20:53592377-53594956 occupies the top position in terms of significance across the bone marrow of different pediatric AML patients and AML cell lines. This genomic locus corresponds to the location of the ZNF217 gene **(Figure 1A, Figure S1 A-D, Figure S2 A-B, Table S7)**. Analysis using the SangerBox database indicated that ZNF217 expression is significantly elevated in AML **(Figure 1B)**, then we categorized 142 patients with AML into “high ZNF217 expression” and “low ZNF217 expression” groups based on the median expression value and subsequently compared their survival differences, the results imply that the low ZNF217 expression group might have a better prognosis. Nevertheless, the P-value of 0.09, which is close to the threshold for significance, suggests that this difference is not statistically significant, possibly due to the limited sample size **(Figure 1C)**. As a result, we focused our further investigations on a more detailed analysis of ZNF217.

We also demonstrated using RNA-Seq and qRT-PCR that ZNF217 expression was significantly higher in pediatric AML patients than in normal pediatric HSPCs (GSE190775^7^ and GSE260775) **(Figure 1D-E)**. To explore how AML-related SEs may regulate ZNF217, we analyzed THP-1 HiC data and H3K27ac ChIP-seq data from pediatric AML samples, as well as from the MV4-11 and THP1 cell lines. Our goal was to identify interactions between SEs and the ZNF217 promoter regions in AML. The visualization showed that the identified SEs interacted with the ZNF217 promoter** (Figure 1F, Figure S3)**, indicating that ZNF217 may be a target gene regulated by these SEs. We performed an integrated analysis of THP-1 HiC and H3K27ac ChIP-seq data from pediatric AML samples, along with data from the MV4-11 and THP1 cell lines.

To further validate this hypothesis, we randomly selected three component enhancers (E1, E3, and E5) from the identified SEs. Additionally, we chose the sequence of the RUNX1 super-enhancer as a positive control. We then cloned all these sequences and inserted them into the pGL3 promoter luciferase reporter vector 37. Subsequently, we transfected the constructed luciferase reporter vectors into MV4-11 cells. Our observations showed a significant increase in activity for all three ZNF217 enhancers compared to the control (Ctrl), with enhancer E1 exhibiting the highest activity **(Figure S4)**.

### ZNF217, ELF1, MEF2D, RUNX2, and FOXP1 are integral components of the AML CRC

After demonstrating that ZNF217 is regulated by SEs, we aimed to identify the upstream TFs that modulate its activity. We utilized the H3K27ac ChIP-seq dataset from 11 pediatric AML patients, as reported in our previous study (GSE188605 7). Using this data, we examined the transcriptional connections among TFs with a CRC model 35 **(Table S8-Table S18)**. We successfully identified a group of candidate TFs, namely, ZNF217, RREB1, RARA, RUNX2, ZBTB7A, ERG, MEF2D, ELF1, FOXP1, CIC, ETV6, JUNB, and IRF1. These candidate TFs exhibited higher predicted transcriptional connectivity compared to the remaining transcription factors.

**Figure 2:**
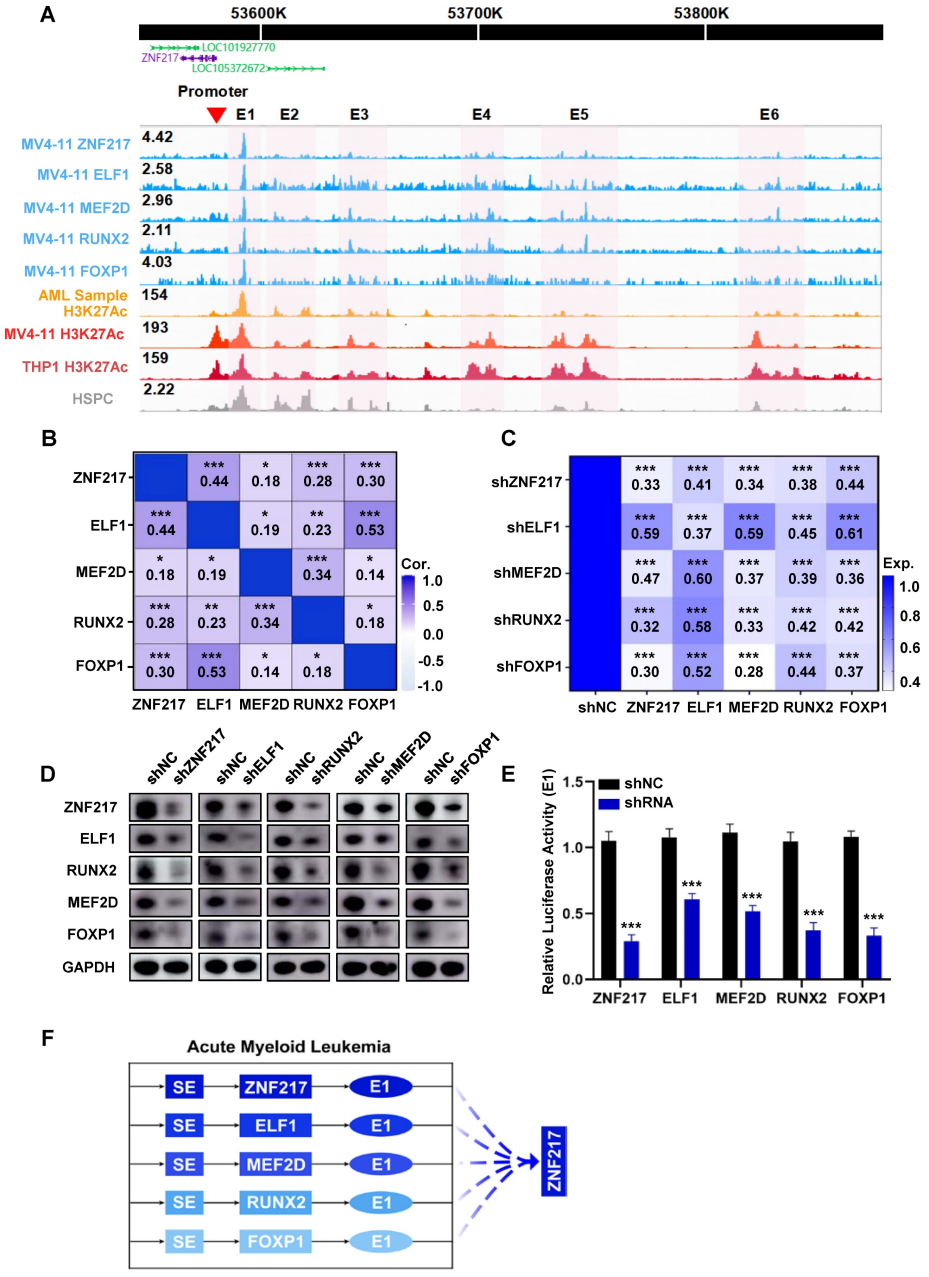
** ZNF217, ELF1, MEF2D, RUNX2, and FOXP1 co-occupy the SE region of ZNF217 to regulate its expression. A.** A WashU plot of CUT&Tag data illustrates the co-occupancy (shaded) of ZNF217, ELF1, MEF2D, RUNX2, and FOXP1 at the SEs regions of ZNF217. **B.** A heatmap was generated to depict the Pearson correlation coefficient between candidate TFs in AML (n=173). **C.** A heatmap was created to display the fold changes in mRNA levels of candidate TFs after knockdown of each TF using shRNA in MV4-11 cell line. **D.** Western blotting analysis was performed to validate the coregulation among candidate TFs in MV4-11 cells. **E.** The luciferase activity of E1 were measured following shRNA knockdown of each candidate TF in MV4-11 cells. **F.** A schematic diagram depicts the mode of co-regulation of ZNF217 by the five candidate TFs through enhancer1 (E1).

Subsequently, we performed CUT&Tag experiments on these TFs and successfully acquired data for ZNF217, RUNX2, MEF2D, ELF1, and FOXP1** (Table S19-Table S24, Figure S5)**. Our analysis revealed shared occupancy regions among ZNF217, ELF1, RUNX2, MEF2D, and FOXP1, as identified through metagene analysis, highlighting their robust cooperative interaction **(Figure 2A, Figure S6)**. To validate this finding, we queried the database (Sangerbox) and discovered a positive pairwise correlation among the expression levels of ZNF217, ELF1, RUNX2, MEF2D, and FOXP1 in AML** (Figure 2B, Figure S7)**. Additionally, we used shRNA to individually silence each of these TFs in MV4-11 cells. Interestingly, knocking down any of these genes significantly reduced the expression levels of the other four TFs at both the mRNA and protein levels** (Figure 2C-D)**.

Then we incorporated H3K27ac ChIP-Seq data acquired from pediatric AML samples, MV4-11 cells, and THP1 cells. Our analysis of CUT&Tag data, along with the visualization of results, revealed that the SE region of ZNF217 in MV4-11 cells was co-occupied by all the five TFs **(Figure 2A)**. After disrupting ZNF217, ELF1, RUNX2, MEF2D, and FOXP1 in MV4-11 cells, we observed a significant decrease in E1 activity **(Figure 2E)**. In addition, Co-IP experiments showed that ZNF217 in MV4-11 cells interacted with the ELF1, MEF2D and LDB1 proteins **(Figure S8)**, providing protein-level evidence of their potential cooperation. This indicates that these five TFs regulate ZNF217 expression by influencing E1 activity **(Figure 2F)**.

### The function of ZNF217 in AML

**Figure 3:**
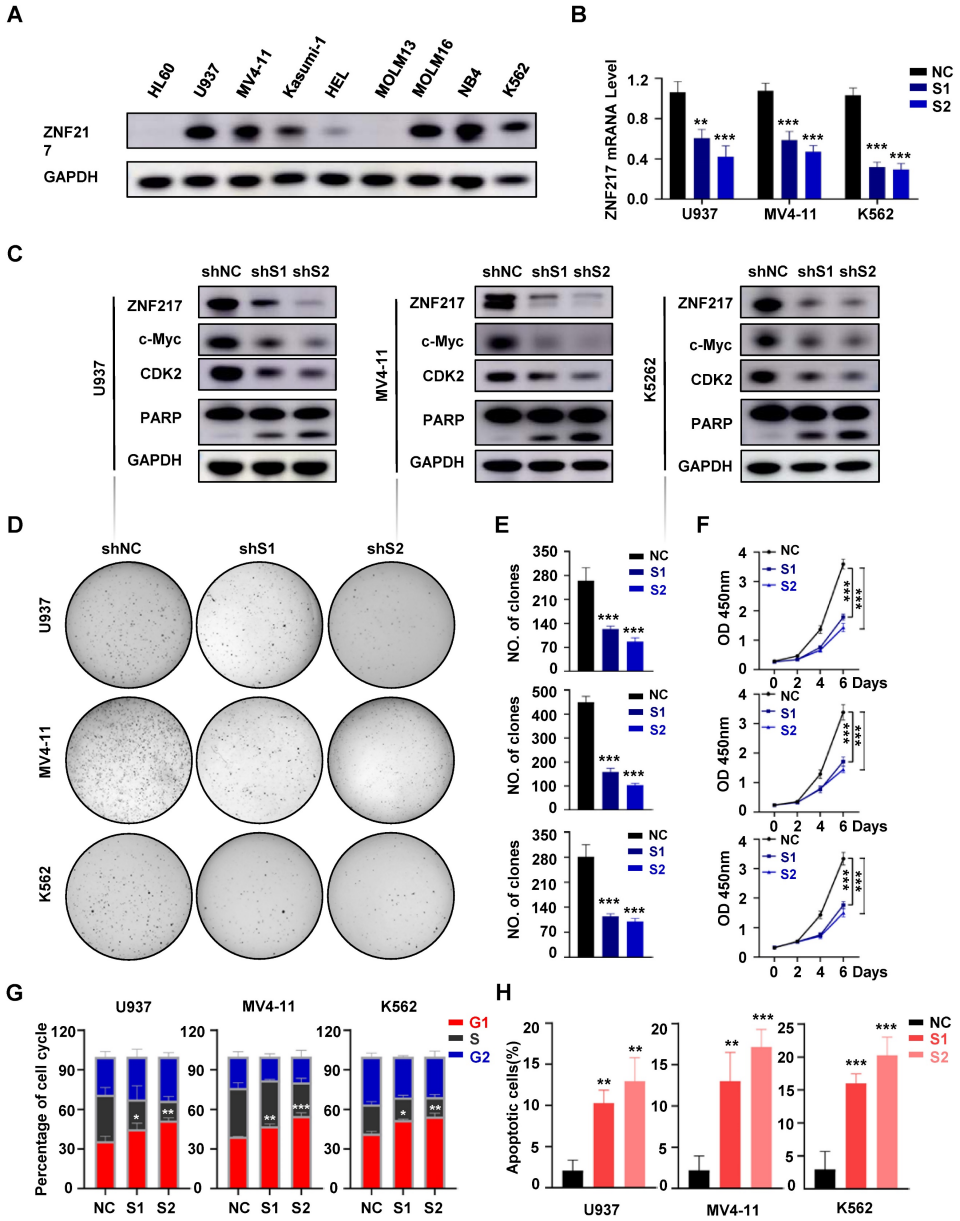
** Knockdown of ZNF217 suppresses AML proliferation *in vitro*. A.** The expression of ZNF217 was assessed in AML cell lines using Western blotting. **B.** The efficiency of ZNF217 knockdown in AML cells was evaluated by qRT-PCR. **C.** The expression levels of ZNF217, c-Myc, CDK2, PARP, and GAPDH in AML cells were examined by Western blotting analysis. **D-E.** Colony formation test was carried out to evaluate the impact of ZNF217 on colony formation. **F.** The CCK8 assay was used to determine the proliferation of AML cells transfected with sh-NC or sh-ZNF217. **G.** Knockdown of ZNF217 led to block cell cycle at the G1 phase in AML cells. **H.** Knockdown of ZNF217 led to an increase in apoptotic rates in AML cells.

To elucidate the function of ZNF217 in AML, we evaluated its protein expression in various AML cell lines** (Figure 3A)**. For *in vitro* functional evaluation, we selected the U937, MV4-11, and K562 cell lines. We designed two distinct shRNA vectors, sh-ZNF217#1 and sh-ZNF217#2, targeting different regions of ZNF217. Compared with the negative control (sh-NC), both sh-ZNF217#1 and sh-ZNF217#2 effectively reduced ZNF217 expression at both mRNA and protein levels in AML cell lines** (Figure 3B-C)**. Additionally, *in vitro* experiments showed that ZNF217-knockdown AML cells had fewer cells, irregular sizes, and increased lysis** (Figure S9A)**. Importantly, cell colony formation and proliferation were significantly inhibited** (Figure 3D-F)**, leading to G1 arrest **(Figure 3G, Figure S10A)** and increased apoptosis **(Figure 3H, Figure S10B)**, suggesting that ZNF217 plays a vital role in AML cell survival *in vitro*.

We further evaluated the role of ZNF217 in healthy human HSPCs by isolating CD34^+^CD38^-^ cells from healthy donors via flow cytometry and performing lentivirus-mediated silencing to decrease ZNF217 expression** (Figure S11A-B)**. Flow cytometric analysis confirmed a significant reduction in ZNF217 expression, with no significant alterations in the proliferation rate or colony-forming ability of HSPCs **(Figure S11C-D)**.

**Figure 4:**
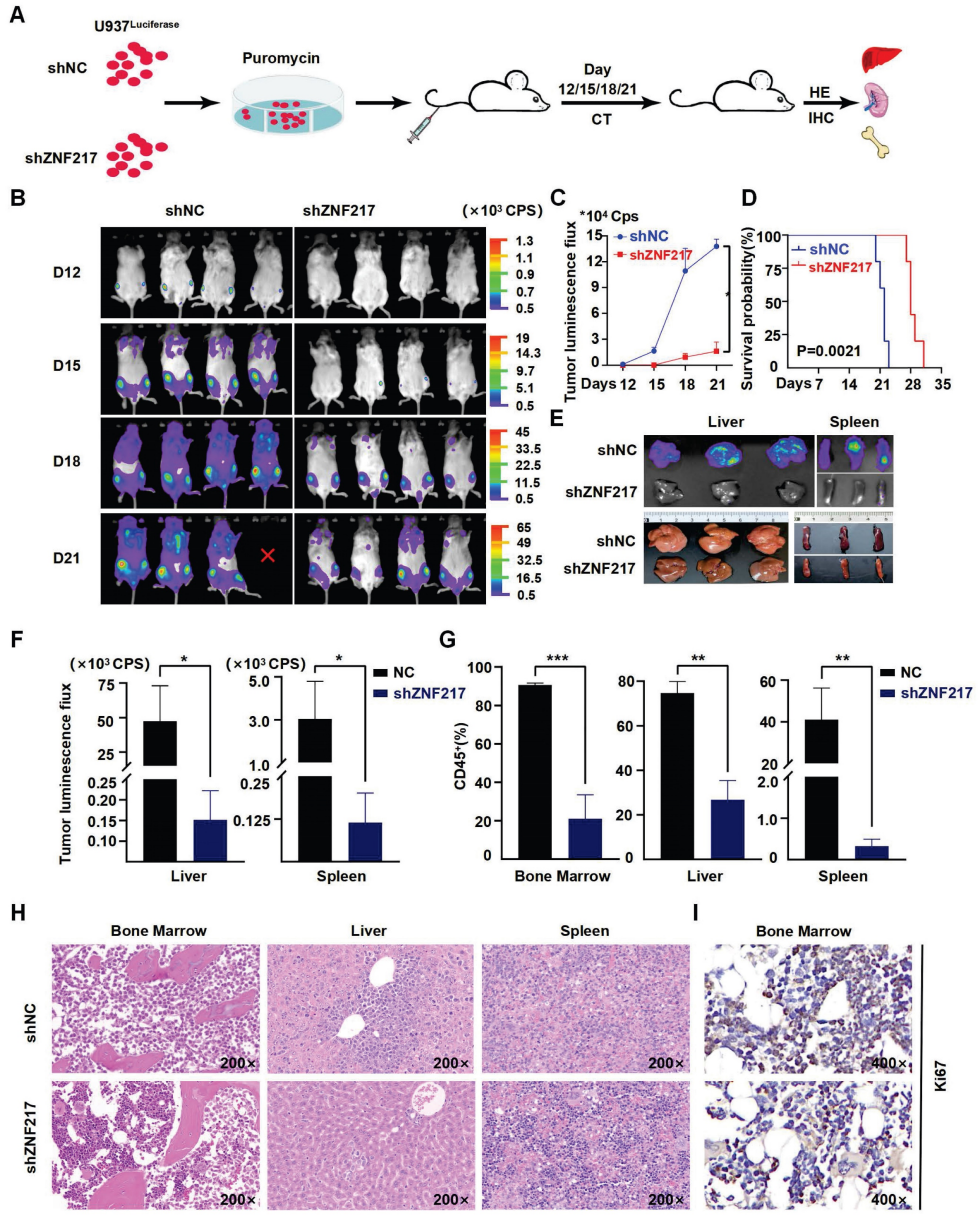
** Knockdown of ZNF217 suppresses AML proliferation *in vivo*. A.** Schematic diagram illustrating the animal experimental model. **B-C.** Representative bioluminescence imaging and statistical analysis at various time points comparing ZNF217 knockdown group with the control group. **D.** The survival curves were presented in the two groups of mice. **E-F.** Bioluminescence imaging and statistical analysis of liver/spleen in ZNF217 knockdown group in comparison to the control group. **G.** Comparison of the percentage of CD45^+^ cells in bone marrow/liver/spleen tissues between ZNF217 knockdown group and the control group. **H.** HE staining assessment of bone marrow, liver, and spleen in the two groups of mice. **I.** Representative images of IHC staining (Ki67) in mice bone marrow.

To validate the impact of ZNF217 on AML cell survival *in vivo*, we used an NSG mouse model of AML. Initially, we labeled U937 cells with luciferase and then injected either ZNF217-silenced or negative control U937-luciferase cells (1×10^6 cells) into the tail veins of the mice **(Figure 4A)**. Notably, the sh-ZNF217 group showed a significant reduction in fluorescence signal intensity compared to the control group** (Figure 4B-C)**, along with a decrease in fluorescence intensity in both the liver and spleen **(Figure 4E-F)** and discernible reductions in the proportions of bone and liver/spleen CD45+ cells** (Figure 4G, Figure S9B)**, indicating a reduced leukemia burden in the sh-ZNF217 group. In addition, the sh-ZNF217 group displayed a significantly increased survival time** (Figure 4D)**. Furthermore, HE staining and immunohistochemical analyses of the bone, liver, and spleen tissues revealed a notable reduction in tumor cell infiltration** (Figure 4H)** and decreased expression of ZNF217, Ki67, and C-MYC in bone/liver/spleen tissues** (Figure 4I, Figure S12A-C)**. These findings further support the conclusion that silencing ZNF217 may attenuate tumor progression *in vivo*.

### MYB is an important downstream molecule of ZNF217

**Figure 5:**
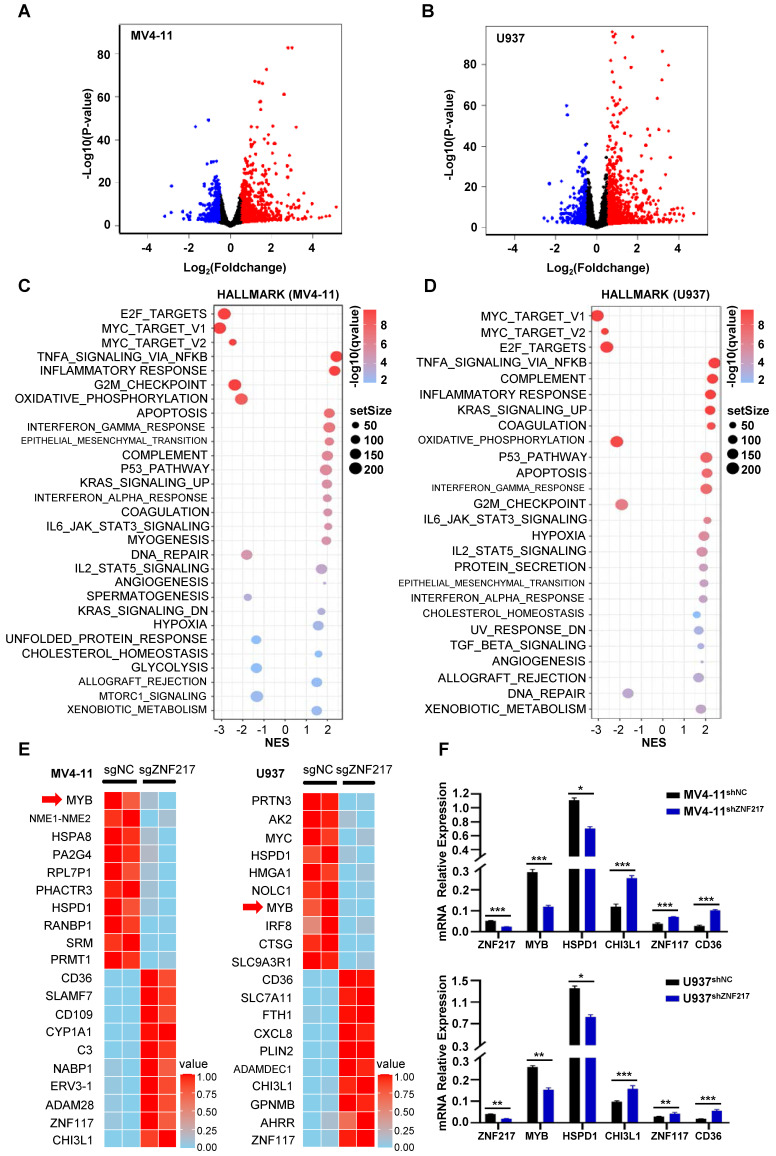
** Changes in gene expression following ZNF217 knockout. A-B.** RNA-Seq analysis, depicted via volcano plot, revealed the differential expression of genes in MV4-11 and U937 cells between the ZNF217 knockout group and the control group. **C-D.** GSEA analysis of Hallmark pathways revealed significant enrichment of differentially expressed genes in both cell lines, particularly within signaling pathways associated with MYC, showing negative NES scores indicative of downregulation. **E.** The heatmap illustrates the top differentially expressed genes in MV4-11 and U937 cells subjected to ZNF217 knockout. **F.** The bar graph depicts changes in mRNA levels of ZNF217, MYB, HSPD1, CHI3L1, ZNF117, and CD36 subsequent to ZNF217 silencing in MV4-11 and U937 cell lines.

Silencing ZNF217 significantly inhibited the proliferation of AML cells, and this inhibition was associated with alterations in the transcriptional regulatory network. The CRISPR-Cas9 system was used to knockout ZNF217 in U937 and MV4-11 cells. A comparative analysis of RNA-Seq data from the control and ZNF217-knockout groups identified 1,285 differentially expressed genes in U937 cells (873 upregulated and 412 downregulated) and 1,433 in MV4-11 cells (909 upregulated and 524 downregulated) (|log2FoldChange| > 0.5 and p < 0.05)** (Figure 5A-B, Table S25-Table S26)**. GSEA analysis of Hallmark pathways showed significant enrichment of differentially expressed genes in both cell lines, especially in signaling pathways linked to MYC, which exhibited negative NES scores indicating downregulation** (Figure 5C-D, Figure S13A-H, Tables S27-Tables S28)**.

A heatmap was generated to display the top ten differentially expressed genes in both cell lines, ranked by P value. Identifying the overlap of these genes revealed common genes including MYB, HSPD1, CHI3L1, ZNF117, and CD36. Notably, MYB and HSPD1 were identified as downregulated genes** (Figure 5E)**. qRT-PCR validation in ZNF217-knockdown U937 and MV4-11 cells confirmed the consistency of the trend observed in the RNA-Seq data **(Figure 5F)**.

**Figure 6:**
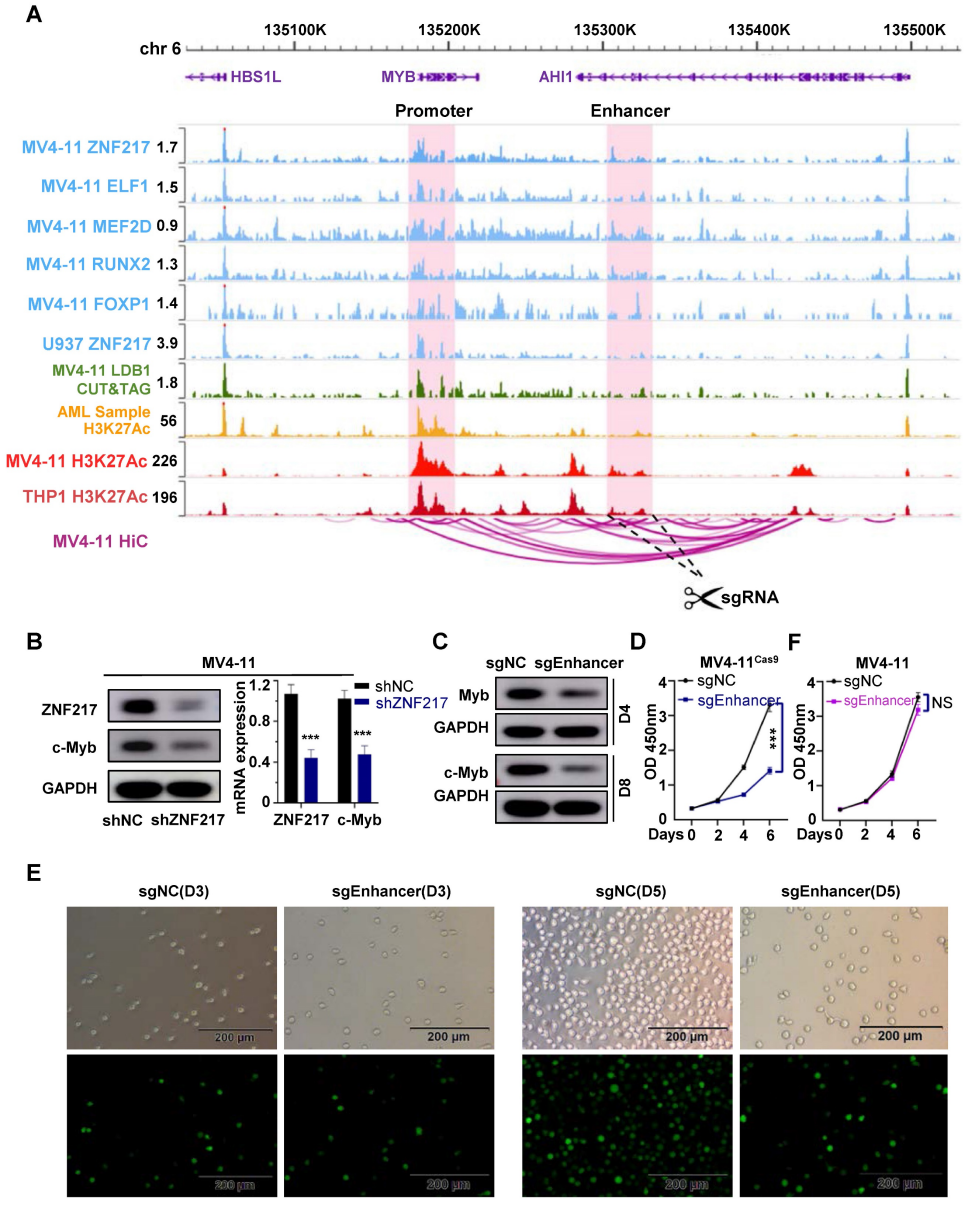
** MYB is identified as a key downstream target gene of ZNF217 in AML cells. A.** WashU plot visual analysis highlights the binding sites (shaded) of ZNF217, ELF1, MEF2D, RUNX2, and FOXP1, while Hi-C analysis reveals their interaction relationships in MV4-11 cell line. **B.** In MV4-11 and U937 cells, interfering with ZNF217 resulted in decreased protein and mRNA expression of MYB. **C.** Knockout of the identified enhancer sequence led to decreased MYB protein expression. **D.** Knocking out the enhancer sequence in MV4-11^cas9^ cells impaired cell proliferation. **E.** Results from the white slice experiment show that knocking out the enhancer sequence significantly decreased the MV4-11^cas9^ cells proliferation in comparison to the control group.** F.** Knocking out the enhancer sequence in MV4-11 cells had no remarkable impact on cell proliferation.

In MV4-11 cells, MYB demonstrated the most significant decrease following ZNF217 knockout, with its promoter region concurrently occupied by the five TFs identified in our study **(Figure 6A)**. Similarly, in U937 cells, MYB was ranked 7th among the downregulated genes following ZNF217 knockout (P-value).

We analyzed MYB expression using SangerBox, revealing significantly higher levels in AML patients compared to the normal control group (P<0.05), moreover, patients with low MYB expression had a relatively better prognosis than those with high expression (P=0.08), but the difference is not statistically significant, possibly due to the limited sample size **(Figure S14A)**. Notably, interference with ZNF217 in U937 and MV4-11 cells markedly reduced MYB expression at both mRNA and protein levels** (Figure 6B, Figure S14B)**. Additionally, we observed an interaction between ZNF217 and LDB1 in MV4-11 cells **(Figure S8)**, silencing LDB1 in resulted in a downregulation of MYB protein expression **(Figure S15)**, and by analyzing CUT&Tag data for LDB1 in MV4-11 cells, we successfully identified a regulatory enhancer segment that governs MYB expression** (Figure 6A)**. Using the CRISPR-Cas9 system, we simultaneously deleted the identified enhancer segment in both MV4-11Cas9 and MV4-11 cells. This intervention led to a notable decrease in the protein level of MYB** (Figure 6C)**, thereby resulting in inhibition of cell proliferation **(Figure 6D-E)**. In contrast, these alterations did not affect the proliferation of MV4-11 cells** (Figure 6F)**.

## Discussion

Approximately 30% of all pediatric cancers are leukemias, with AML accounting for 15-20% of these cases. The prognosis for children with AML has improved significantly in recent decades, with long-term survival rates now exceeding 70%. However, the incidence of relapse has remained relatively constant over the past 10-20 years, affecting 24-40% of pediatric patients. Consequently, the long-term survival rate after relapse remains around 30% 37-39. Notably, abnormal transcriptional regulation is a characteristic of AML, and deciphering the central regulatory program in AML could profoundly improve our understanding of its etiology. The transcriptional regulatory activity of SEs in AML has attracted increasing attention 40. The role of MYC, a recognized proto-oncogene, has been broadly investigated in AML. Shi et al. identified an SE cluster located 1.7 Mb from the transcription initiation site of MYC in acute leukemia cells and found that it was occupied by SWI/SNF and the BET protein BRD4 41. Similarly, Roe et al. established the presence of BRD4, the histone acetyltransferase p300/CBP, and assorted AML-related hematopoietic transcription factors (TFs) (PU.1, FLI1, MYB, etc.) in the SE domain of MYC 42. Collective evidence from these studies underscores the involvement of SE regulation in the dynamics of MYC expression and throughout the progression of AML.

In our study, we identified ZNF217 as a frequently regulated gene by SEs in AML. Additionally, ZNF217, along with ELF1, MEF2D, RUNX2, and FOXP1, constitutes CRC in AML. CRC play a crucial role in controlling cell states and determining cell identities, and they exhibit distinct distributions across various cell types and tissues. A classical model for studying these circuits is embryonic stem cells (ESCs), where the core TFs NANOG, POU5F1/OCT4, and SOX2 form an interconnected feedforward loop crucial for establishing and maintaining ESC identity. This loop is also involved in the dedifferentiation of cells into ESC-like induced pluripotent stem cells (iPSCs) 43. CRC are similarly critical in different types of leukemia. J. Ott and colleagues identified a SE-based CRC in chronic lymphocytic leukemia (CLL) involving PAX5, FOXP1, RARA, ETS1, IRF2, and IRF8, highlighting PAX5's dominant role. BET inhibitors can disrupt this SE in CLL, thus inhibiting tumor growth 44. In T-ALL, the transcription factors TAL1, GATA3, and RUNX1 form a positively connected self-regulatory loop that regulates T-cell homeostasis and directly activates MYB to facilitate tumor progression 45. Our data demonstrate that ZNF217 modulates its own transcription by co-occupying its SE with ELF1, MEF2D, RUNX2, and FOXP1, thereby advancing AML progression.

Although there has been little prior research on ZNF217 in AML, we found that ZNF217 expression is higher in AML patient samples than in healthy human HSPCs, additionally, higher ZNF217 expression correlates with a poorer prognosis. Our research also indicates that targeting ZNF217 in AML cell lines causes G1 arrest, increases apoptosis, and decreases proliferation, both *in vivo* and *ex vivo*. Conversely, disrupting ZNF217 expression in healthy human HSPCs did not impact their proliferation or colony-forming ability, highlighting ZNF217's crucial role in AML cell proliferation. Accumulating evidence suggests that ZNF217 contributes to tumorigenesis and disease progression. For instance, overexpression of ZNF217 in ovarian cancer cells accelerates cell proliferation and significantly increases the proportion of cells in the S-phase 46. Additionally, overexpression of ZNF217 in paclitaxel-treated MDA-MB-231 breast cancer cells induces resistance to apoptosis by downregulating pro-apoptotic proteins (Bad, Bak, Bax) and upregulating anti-apoptotic proteins (Bcl-2, Bcl-XL) 47. Notably, a recent study by Adorisio et al. showed that Cucurbitacin D reduces ZNF217 expression in NPM-mutant AML cell lines (OCI-AML3). This treatment inhibits AML cell proliferation by inducing apoptosis and blocking cell cycle progression, which supports our findings 48.

After knockout of ZNF217 in U937 and MV4-11 cells, MYB exhibited the most significant downregulation. Moreover, we identified a proximal enhancer segment near the MYB gene that modulates its expression and demonstrated that deletion of this segment in AML cells leads to decreased MYB expression and reduced proliferation. MYB plays a crucial role in modulating the proliferation and differentiation of hematopoietic stem cells and is a central component of the complex responsible for maintaining aberrant gene expression in various types of leukemia, including AML, CLL, and ALL 44,49. While MYB inhibition has emerged as a potential therapeutic strategy for various leukemias, including AML, the exact mechanism of MYB transcriptional regulation remains unclear.

We identified a protein-protein interaction between LDB1 and ZNF217 in MV4-11 cells. Additionally, we conducted CUT&Tag experiments for LDB1 in the same cell line. LDB1 is an important transcription co-regulator. Research indicates that it interacts with transcription factors like GATA1, ISL1, and LMO2 to form complexes that are essential for erythrocyte differentiation and pancreatic β-cell function 50,51. Our research group has shown that LDB1 acts as an oncogenic transcriptional regulator in AML. We provided evidence that the LDB1-LMO2 protein complex promotes disease progression in AML cells through techniques such as mass spectrometry, co-immunoprecipitation, and CUT&Tag experiments 11. These findings indicate that LDB1 is a central component of the transcriptional regulatory network, forming complexes with various transcription factors to regulate cellular development and function. We then conducted an integrated analysis of CUT&Tag data for LDB1 and five AML-CRC molecules, including ZNF217, alongside H3K27ac ChIP-Seq data from pediatric AML samples and MV4-11/THP1 cell lines, as well as HiC data from MV4-11 cells. This analysis led to the identification of an enhancer that regulates MYB expression. In some studies, the CRISPR-Cas9 technique has been utilized to target BCL11A enhancers in CD34+ cells, which were subsequently reintroduced into patients with β-thalassemia and sickle cell disease, leading to successful treatment 52,53. Here, we propose a potential regulatory mechanism for MYB and provide support for the potential application of the CRISPR-Cas9 technique in AML treatment.

## Conclusion

**Figure 7:**
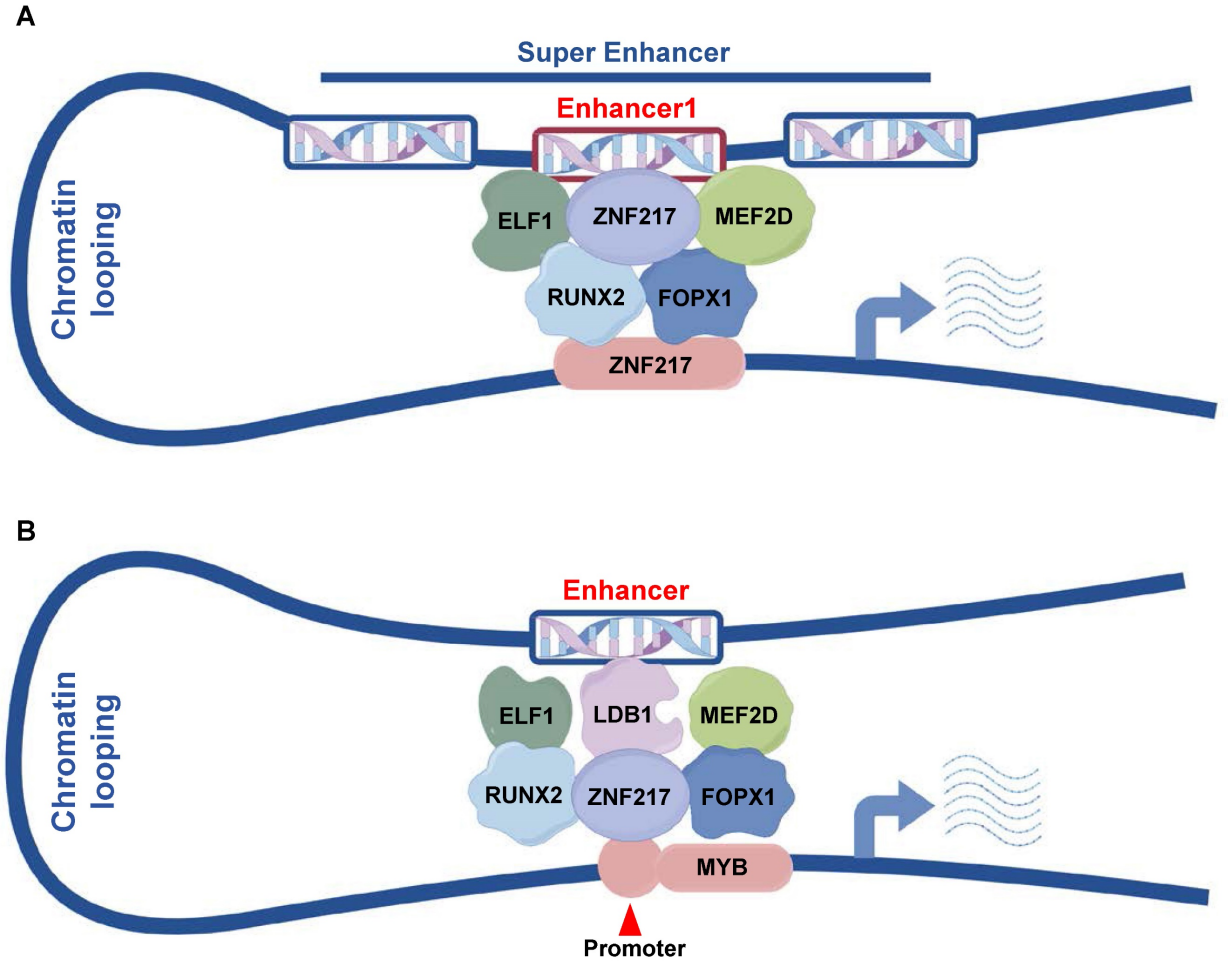
** Pattern diagram. A.** ZNF217, ELF1, MEF2D, RUNX2 and FOXP1 collaboratively regulate ZNF217 expression by co-occupying the enhancer region of ZNF217. **B.** ZNF217, ELF1, MEF2D, RUNX2, FOPX1, and LDB1 engage in collaborative regulation of MYB transcription through the interaction between its promoter and the identified enhancer regions.

In summary, our results identified a new gene regulated by SEs, ZNF217, which is crucial for the proliferation of AML cells. Our mechanistic studies showed that ZNF217, ELF1, MEF2D, RUNX2, and FOXP1 are key components of the AML core regulatory circuit. ZNF217 collaborates with ELF1, MEF2D, RUNX2, and FOXP1 at its own SE to regulate its expression** (Figure 7A)**. Additionally, we demonstrated that MYB is a direct downstream target of ZNF217** (Figure 7B)** and identified a new enhancer that regulates MYB expression. These findings provide valuable insights into the transcriptional regulatory mechanisms that govern AML.

## Supplementary Material

Supplementary figures and tables.

## Abbreviations

TFs: transcription factors
SEs: Super-enhancers
CRC: Core Transcriptional Regulation Circuit
AML: acute myeloid leukemia
shRNA: short hairpin RNA
MM: multiple myeloma
BRD4: bromodomain-containing 4
CDK: cyclin-dependent kinase
T-ALL: T-cell acute lymphoblastic Leukemia
BM: bone marrow
BMMCs: bone marrow mononuclear cells
sgRNAs: Single guide RNAs
sgNC: nontargeting control sgRNA
CLL: chronic lymphocytic leukemia
ESCs: embryonic stem cells
iPSCs: induced pluripotent stem cells
