# Supplementary figures and images for: Core transcriptional regulatory circuitry molecule ZNF217 promotes AML cell proliferation by up-regulating MYB
Source: Int J Biol Sci. 2025 Feb 18;21(5):1966–83. doi: 10.7150/ijbs.103211 (PMC11900805; doi:10.7150/ijbs.103211)

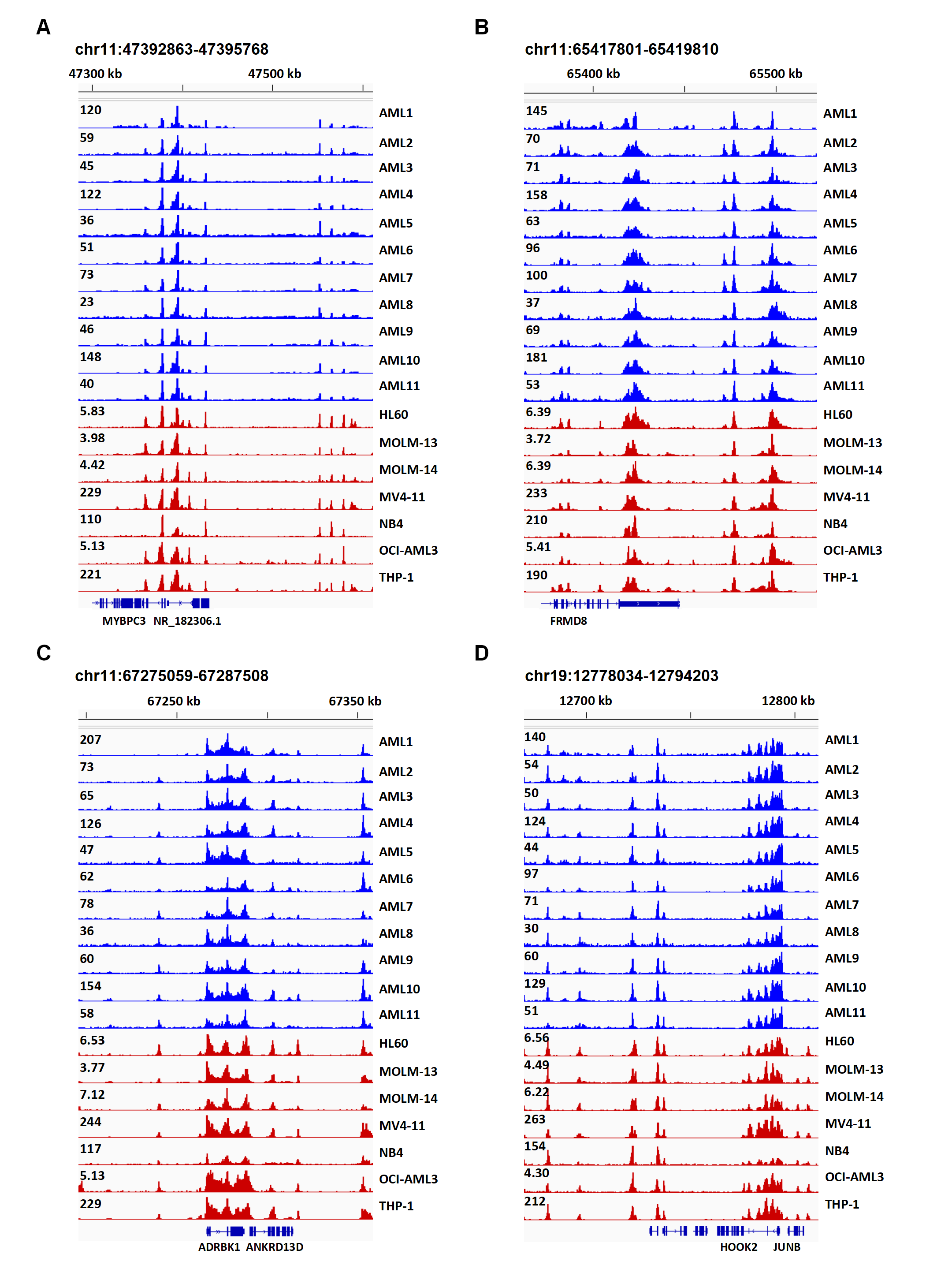

Supplement: Supplementary file 1 — Supplementary figures and tables. [file ijbsv21p1966s1.zip › Supplementary/Figure S1.tif]

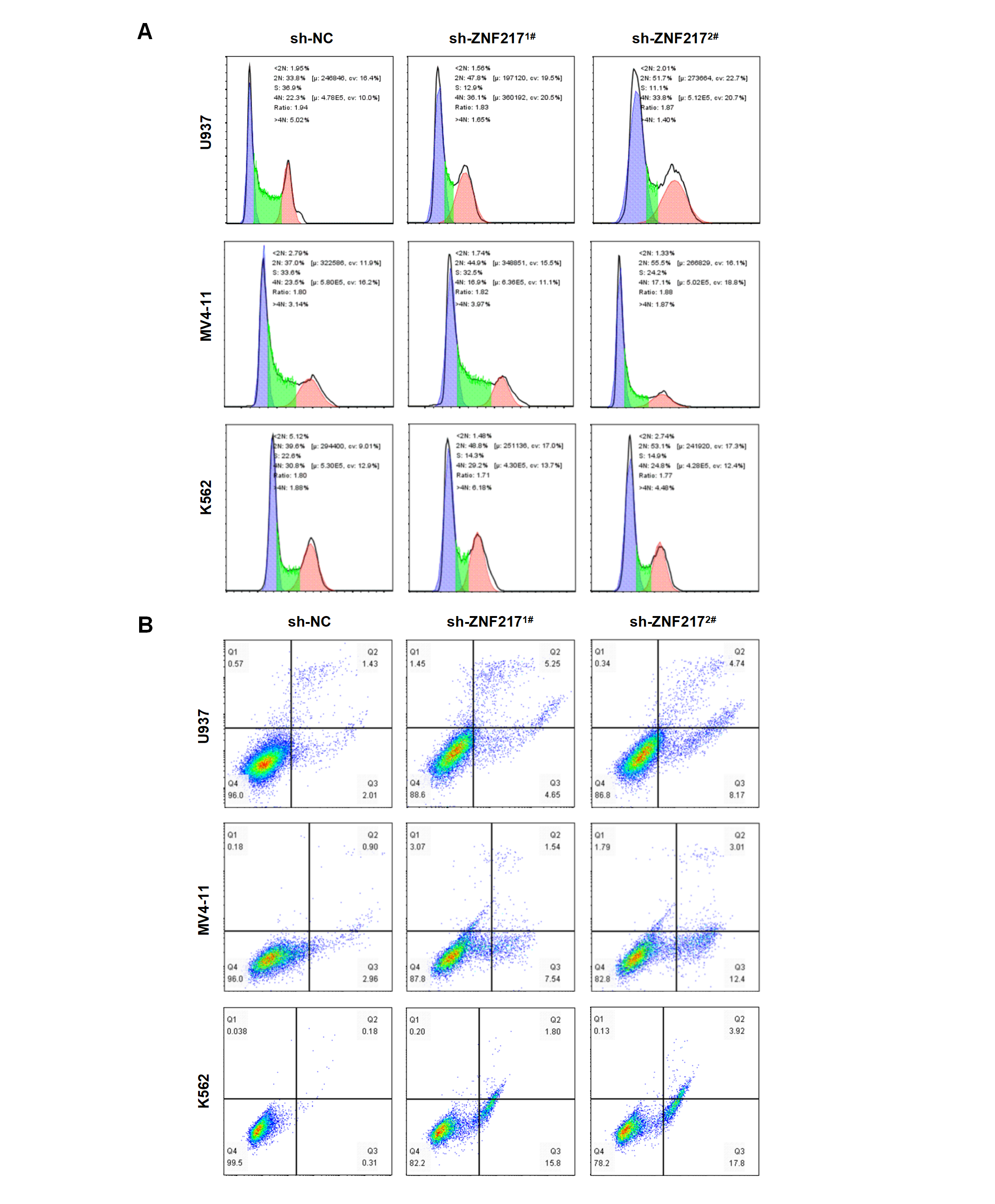

Supplement: Supplementary file 1 — Supplementary figures and tables. [file ijbsv21p1966s1.zip › Supplementary/Figure S10.tif]

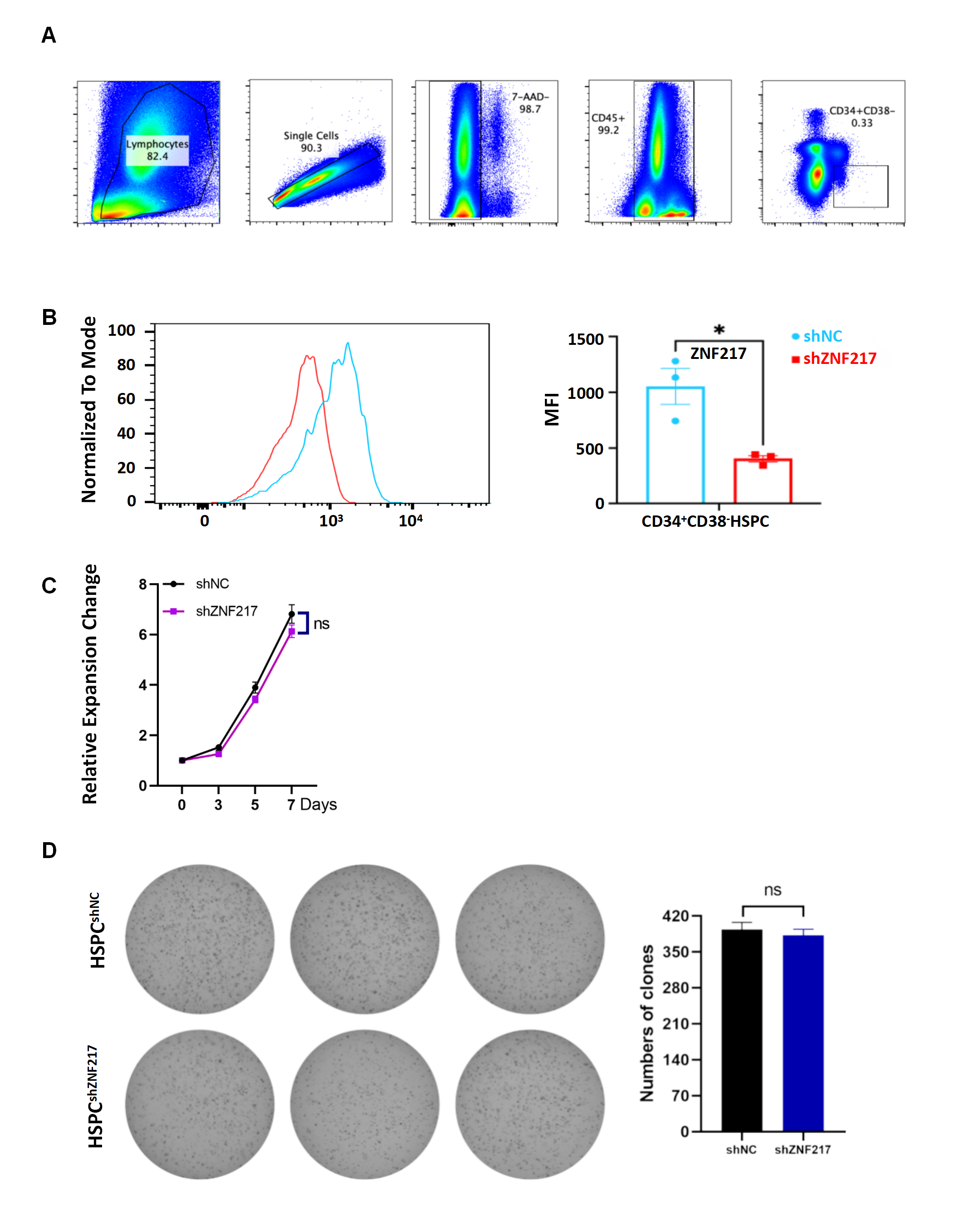

Supplement: Supplementary file 1 — Supplementary figures and tables. [file ijbsv21p1966s1.zip › Supplementary/Figure S11.tif]

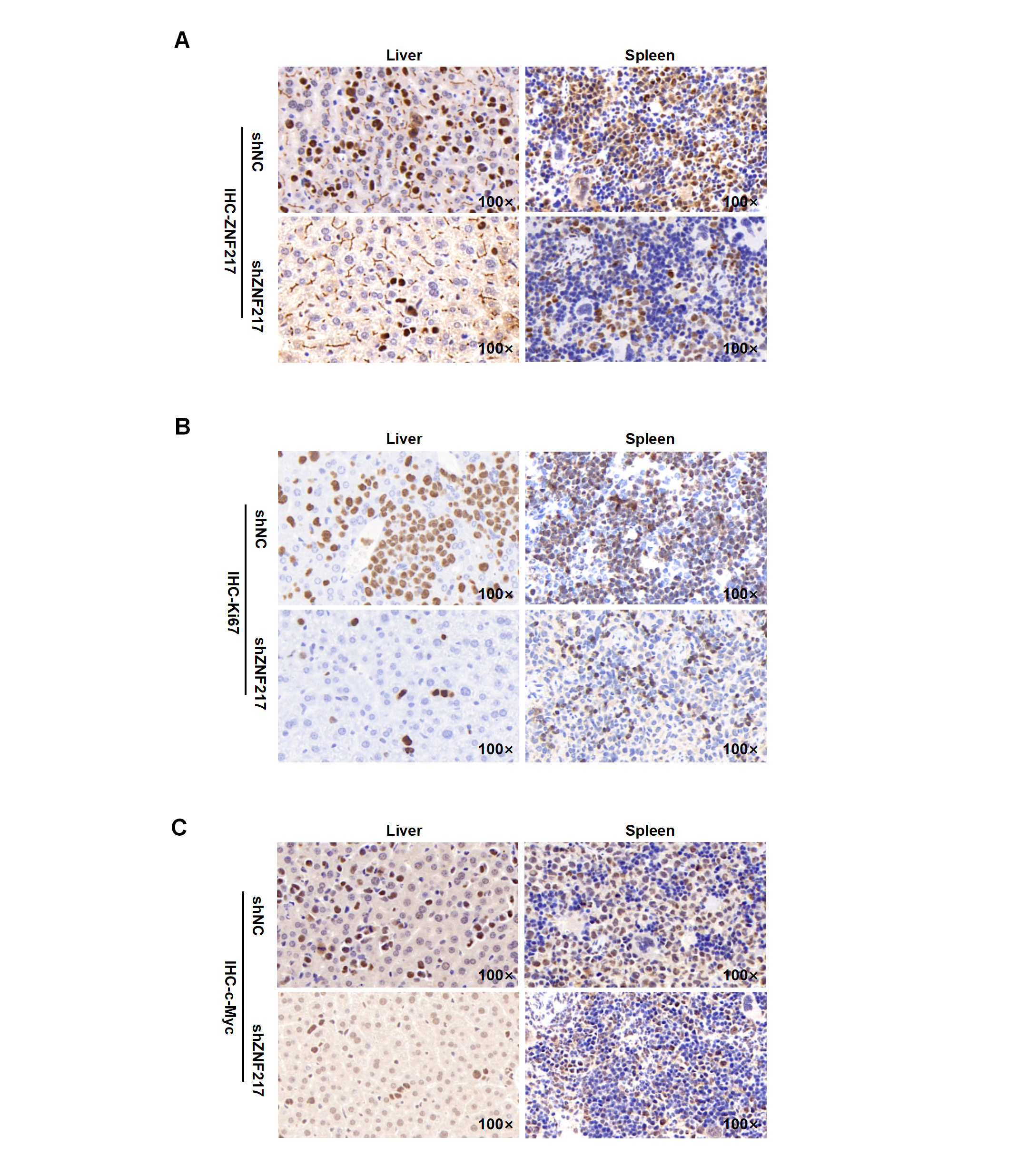

Supplement: Supplementary file 1 — Supplementary figures and tables. [file ijbsv21p1966s1.zip › Supplementary/Figure S12.tif]

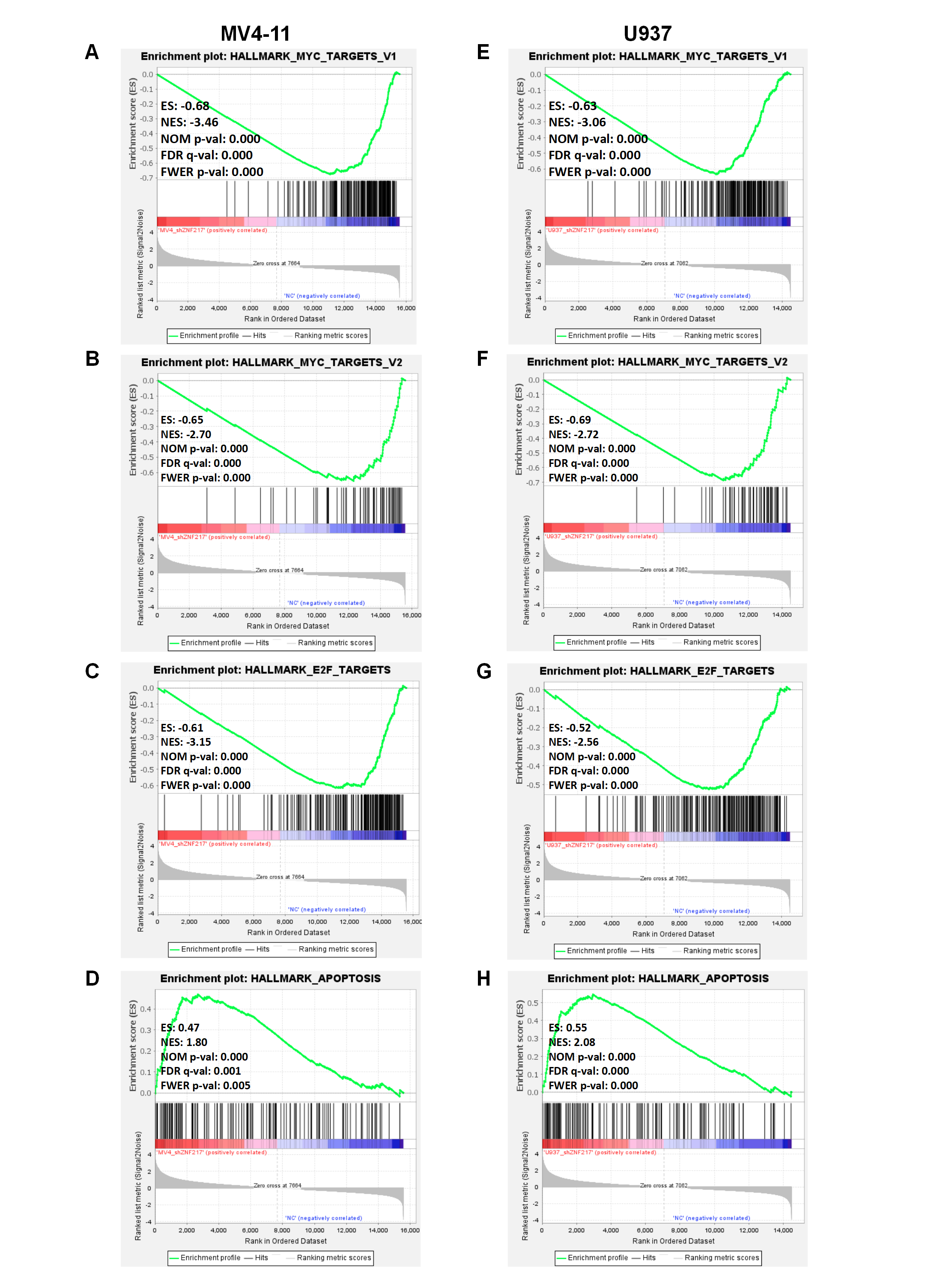

Supplement: Supplementary file 1 — Supplementary figures and tables. [file ijbsv21p1966s1.zip › Supplementary/Figure S13.tif]

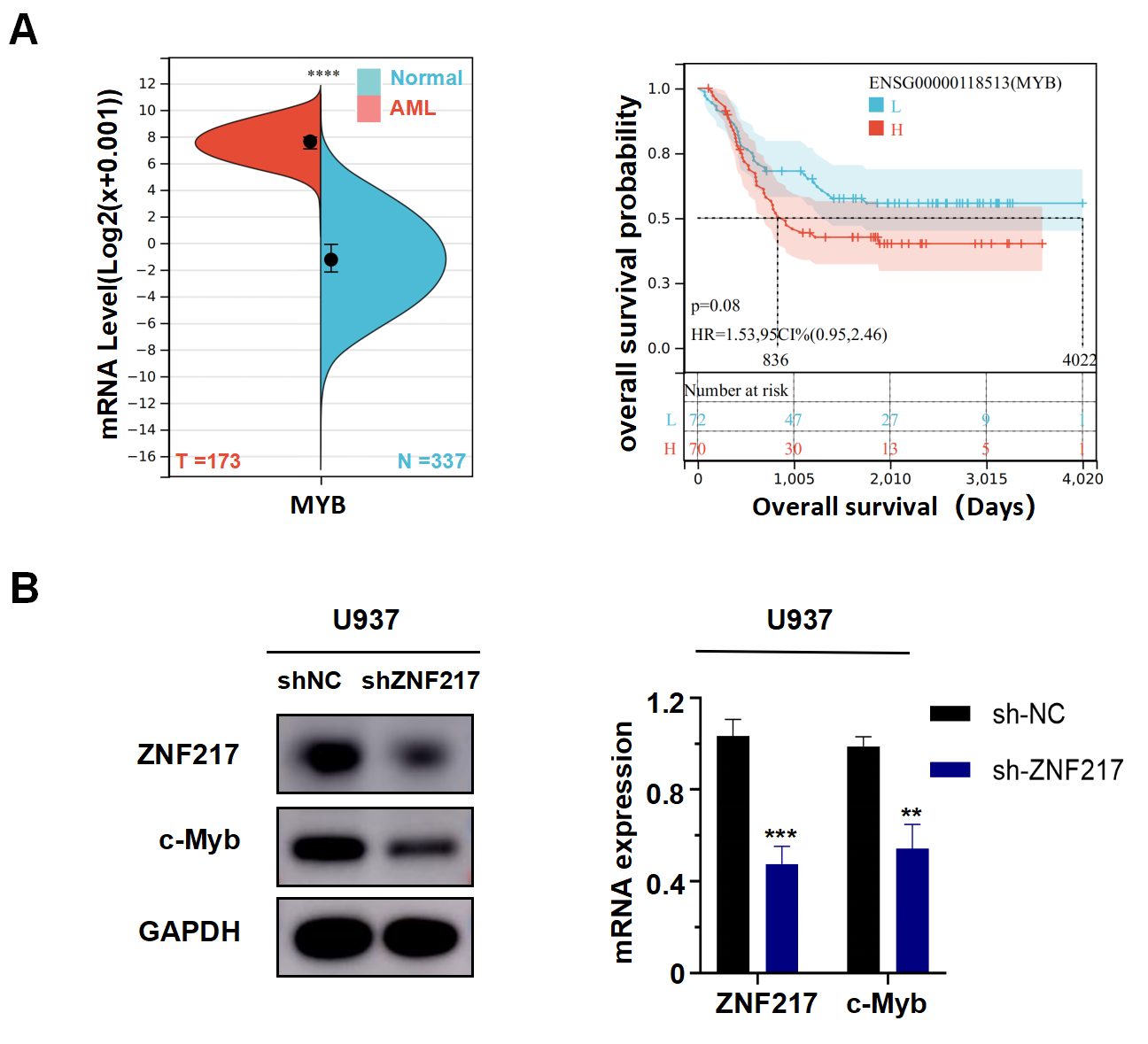

Supplement: Supplementary file 1 — Supplementary figures and tables. [file ijbsv21p1966s1.zip › Supplementary/Figure S14.tif]

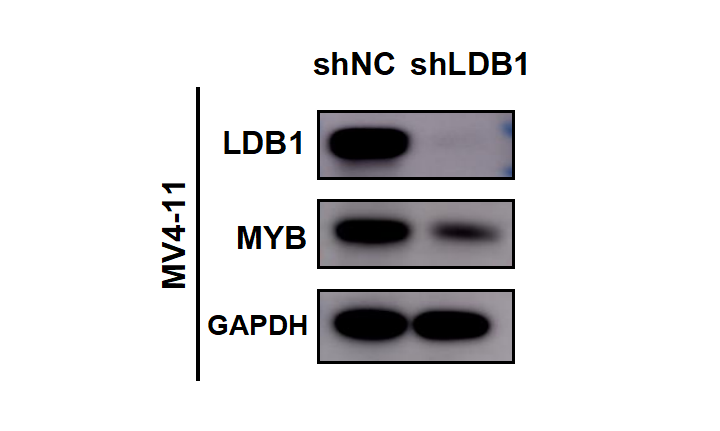

Supplement: Supplementary file 1 — Supplementary figures and tables. [file ijbsv21p1966s1.zip › Supplementary/Figure S15.tif]

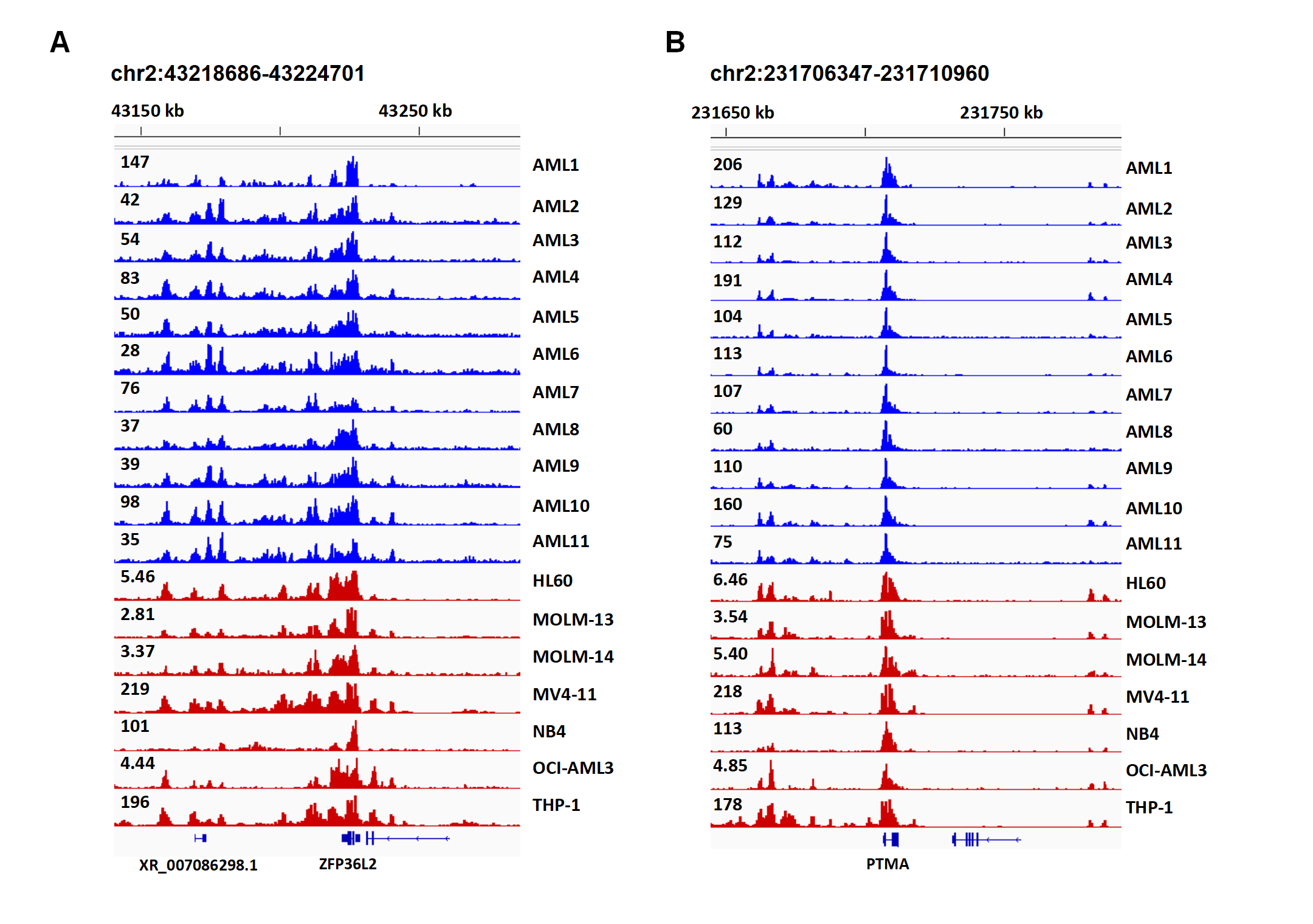

Supplement: Supplementary file 1 — Supplementary figures and tables. [file ijbsv21p1966s1.zip › Supplementary/Figure S2.tif]

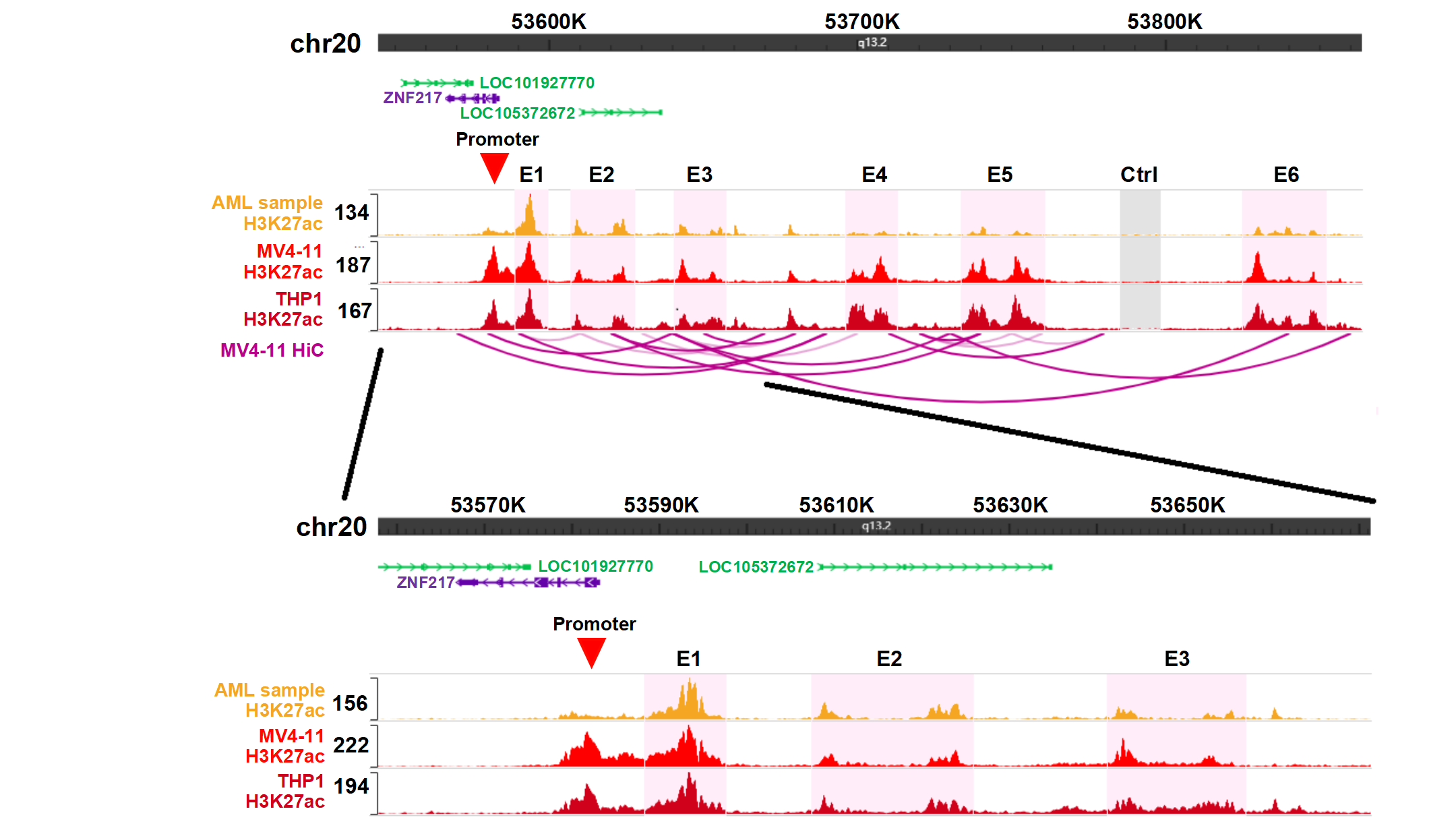

Supplement: Supplementary file 1 — Supplementary figures and tables. [file ijbsv21p1966s1.zip › Supplementary/Figure S3.tif]

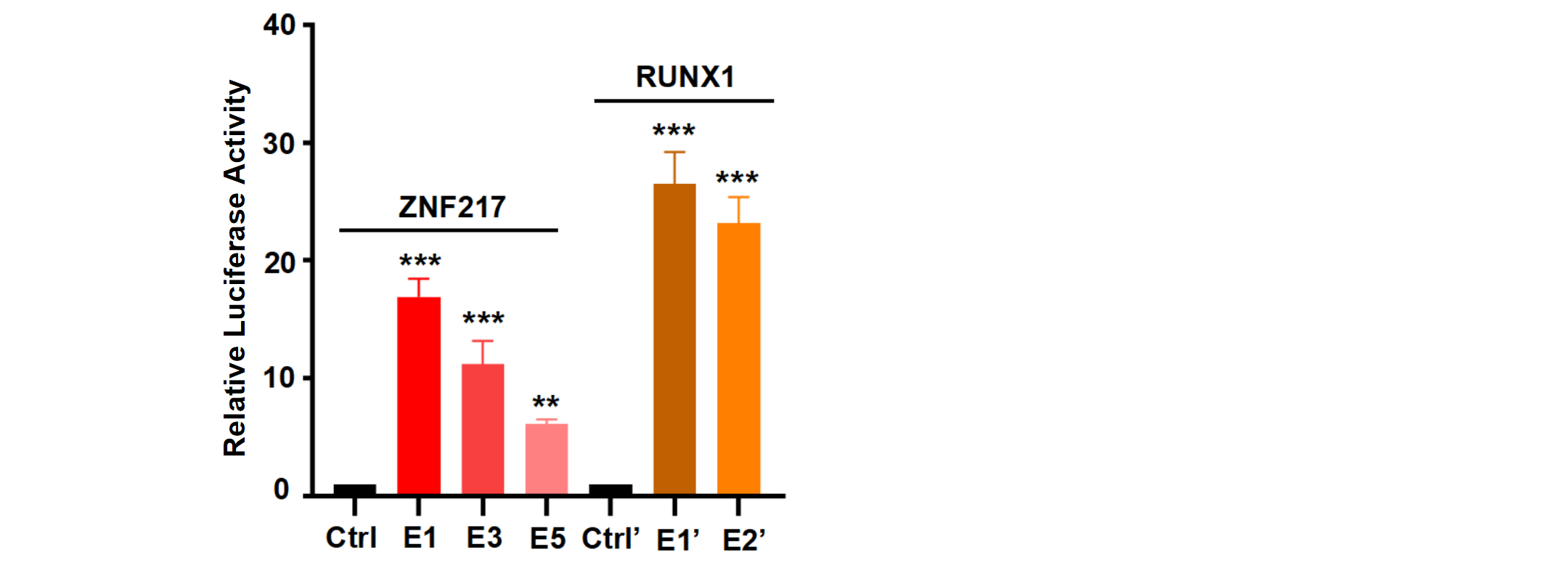

Supplement: Supplementary file 1 — Supplementary figures and tables. [file ijbsv21p1966s1.zip › Supplementary/Figure S4.tif]

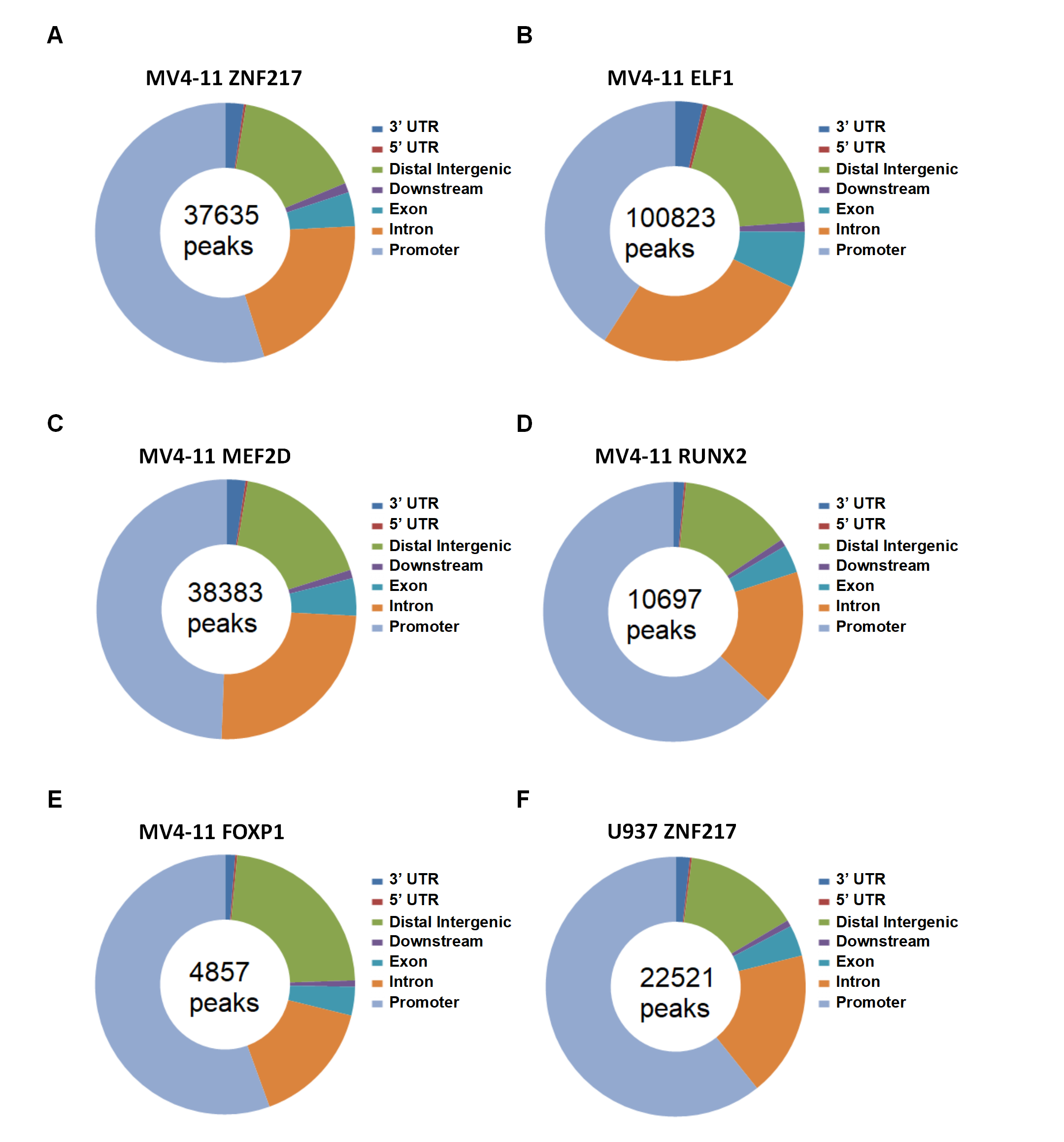

Supplement: Supplementary file 1 — Supplementary figures and tables. [file ijbsv21p1966s1.zip › Supplementary/Figure S5.tif]

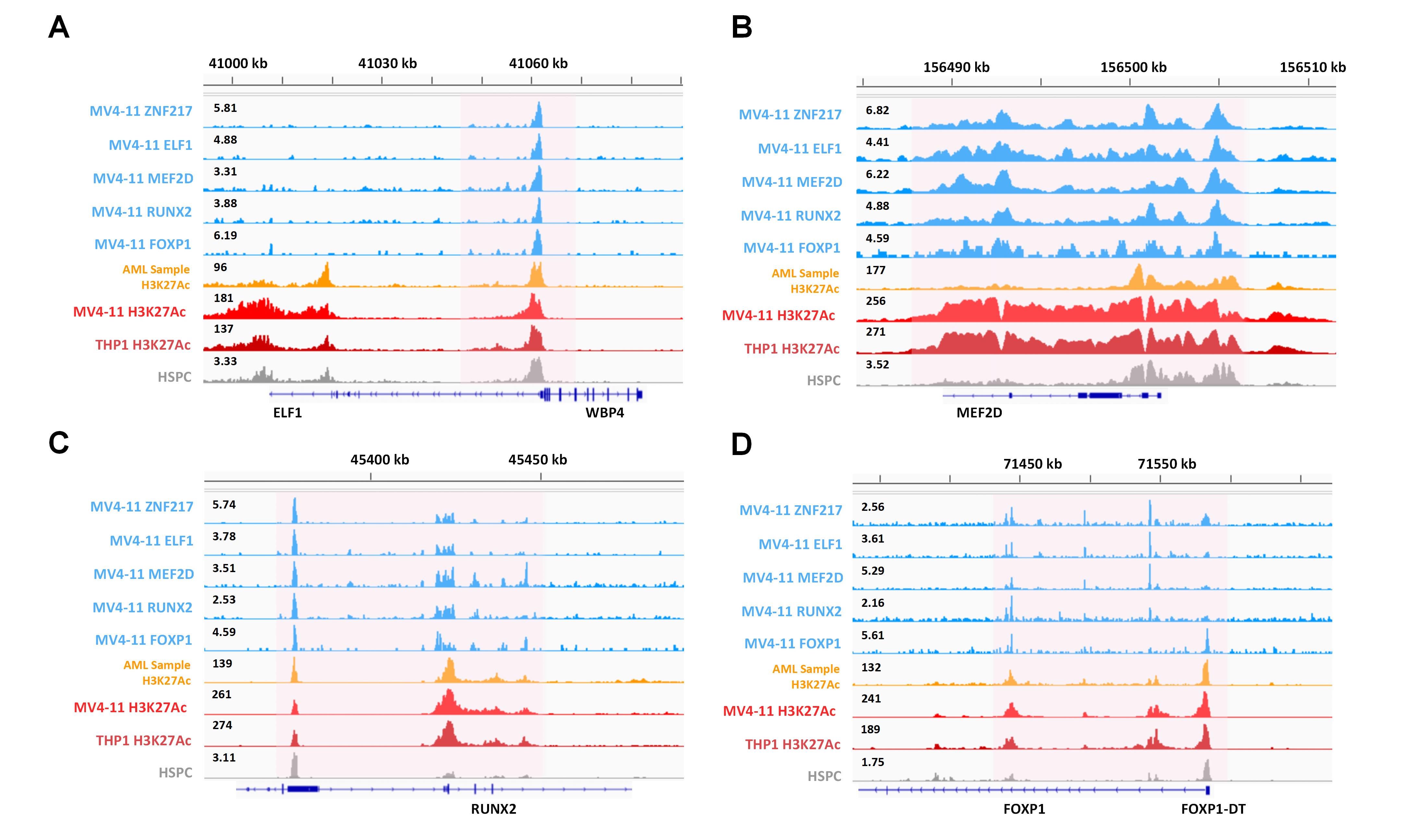

Supplement: Supplementary file 1 — Supplementary figures and tables. [file ijbsv21p1966s1.zip › Supplementary/Figure S6.tif]

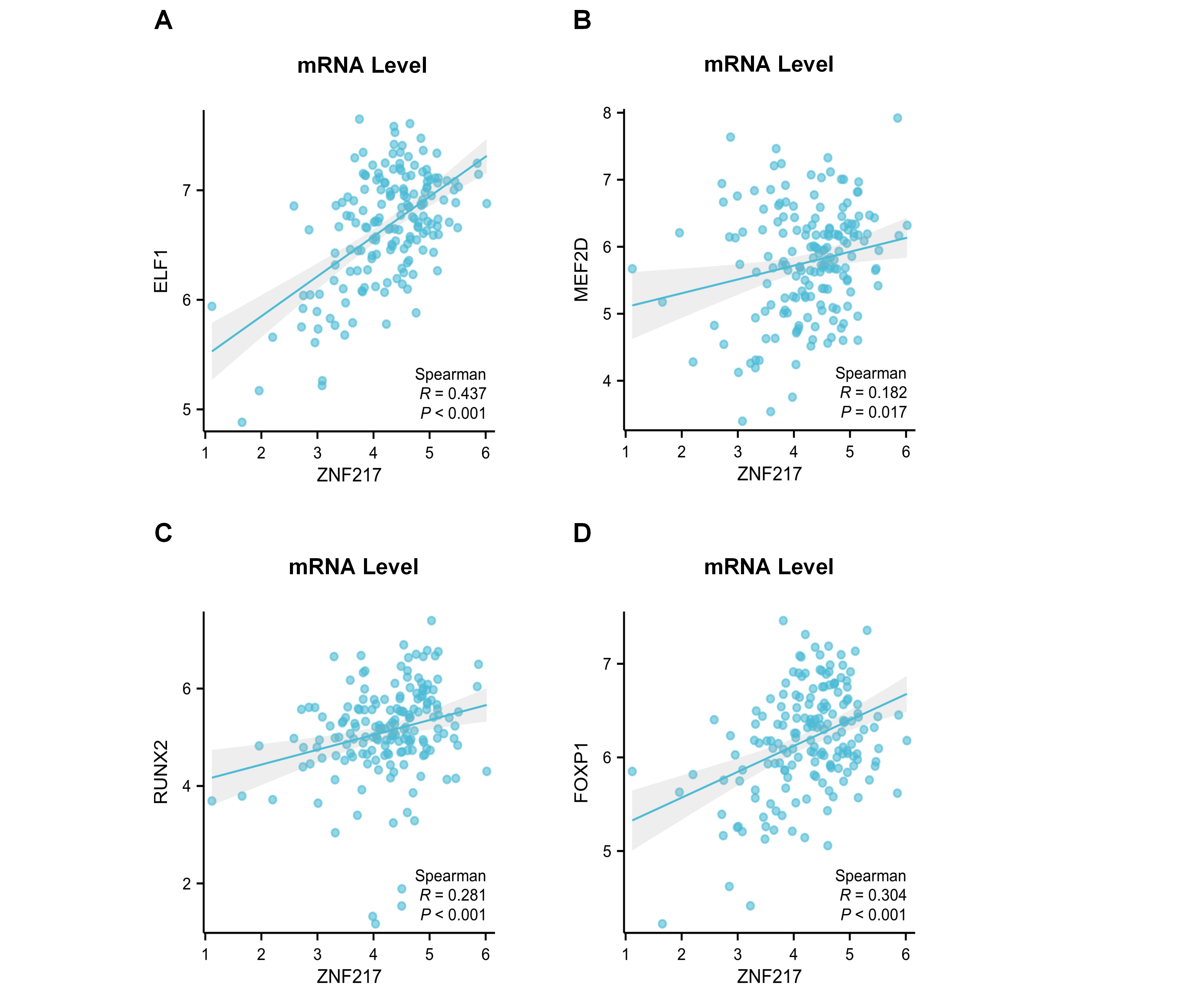

Supplement: Supplementary file 1 — Supplementary figures and tables. [file ijbsv21p1966s1.zip › Supplementary/Figure S7.tif]

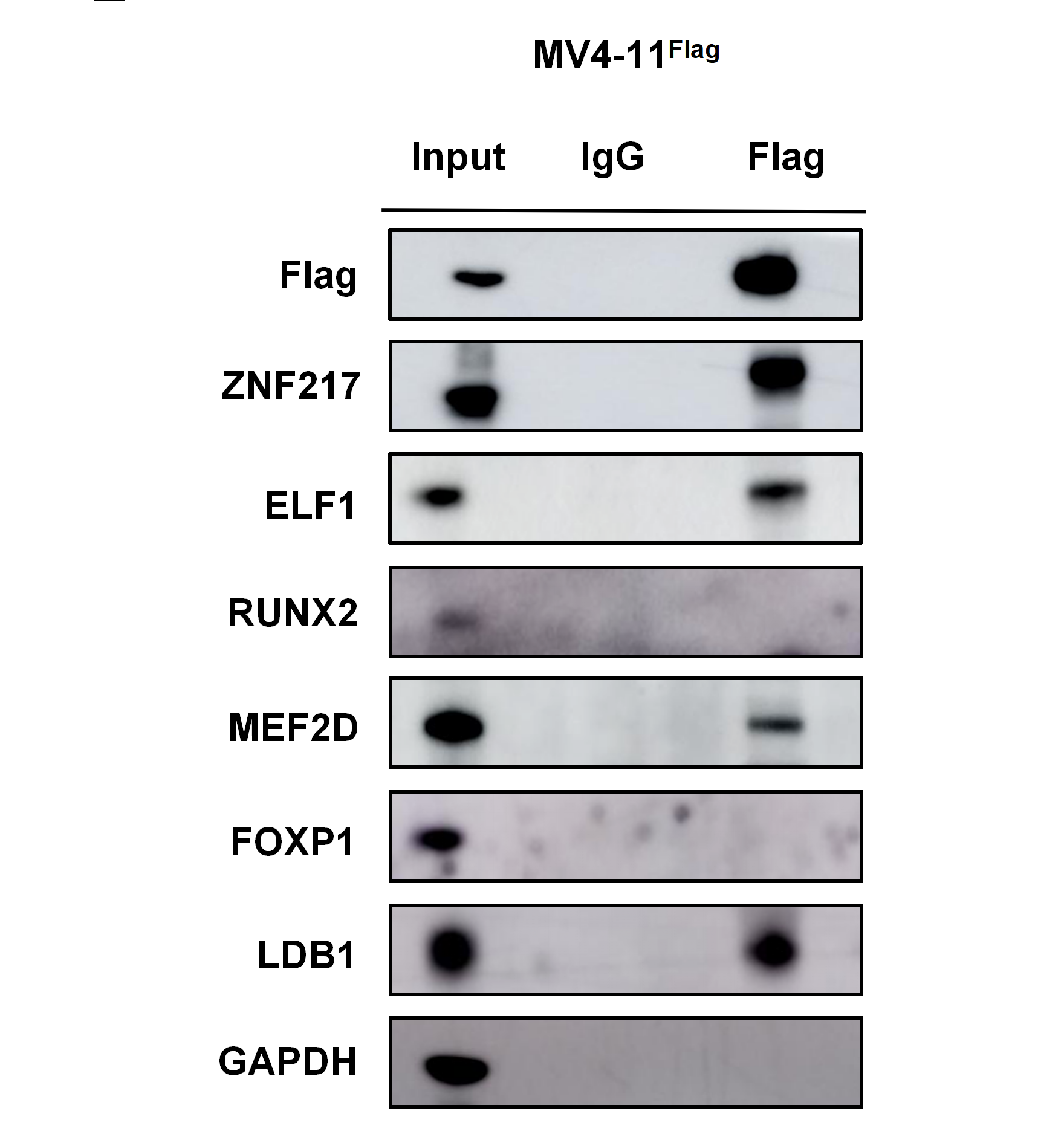

Supplement: Supplementary file 1 — Supplementary figures and tables. [file ijbsv21p1966s1.zip › Supplementary/Figure S8.tif]

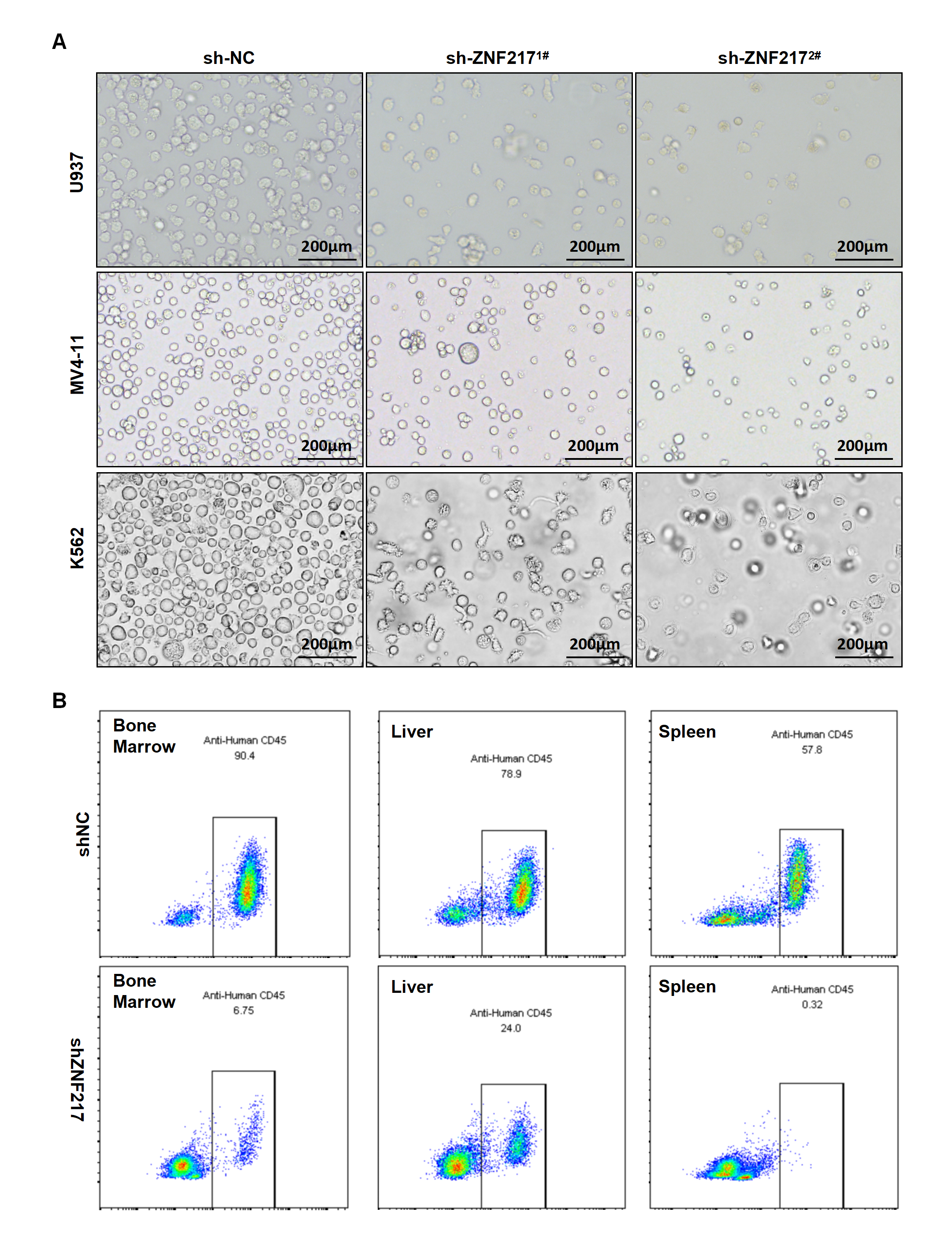

Supplement: Supplementary file 1 — Supplementary figures and tables. [file ijbsv21p1966s1.zip › Supplementary/Figure S9.tif]
